# Integrated bioactive scaffold with aptamer‐targeted stem cell recruitment and growth factor‐induced pro‐differentiation effects for anisotropic meniscal regeneration

**DOI:** 10.1002/btm2.10302

**Published:** 2022-03-03

**Authors:** Hao Li, Tianyuan Zhao, Fuyang Cao, Haoyuan Deng, Songlin He, Jianwei Li, Shuyun Liu, Zhen Yang, Zhiguo Yuan, Quanyi Guo

**Affiliations:** ^1^ Institute of Orthopedics, the First Medical Center, Chinese PLA General Hospital; Beijing Key Lab of Regenerative Medicine in Orthopedics Key Laboratory of Musculoskeletal Trauma & War Injuries PLA Haidian District Beijing China; ^2^ School of Medicine Nankai University Tianjin China; ^3^ Department of Orthopedics the First Affiliated Hospital of Zhengzhou University Eqi District Zhengzhou China; ^4^ Arthritis Clinic & Research Center, Peking University People's Hospital Peking University Beijing China; ^5^ Department of Bone and Joint Surgery, Renji Hospital, School of Medicine Shanghai Jiaotong University Shanghai China

**Keywords:** aptamer, cell recruitment, drug delivery, growth factors, in situ meniscal regeneration

## Abstract

Reconstruction of the knee meniscus remains a significant clinical challenge owing to its complex anisotropic tissue organization, complex functions, and limited healing capacity in the inner region. The development of in situ tissue‐engineered meniscal scaffolds, which provide biochemical signaling to direct endogenous stem/progenitor cell (ESPC) behavior, has the potential to revolutionize meniscal tissue engineering. In this study, a fiber‐reinforced porous scaffold was developed based on aptamer Apt19S‐mediated mesenchymal stem cell (MSC)‐specific recruitment and dual growth factor (GF)‐enhanced meniscal differentiation. The aptamer, which can specifically recognize and recruit MSCs, was first chemically conjugated to the decellularized meniscus extracellular matrix (MECM) and then mixed with gelatin methacrylate (GelMA) to form a photocrosslinkable hydrogel. Second, connective tissue growth factor (CTGF)‐loaded poly(lactic‐*co*‐glycolic acid) (PLGA) nanoparticles (NPs) and transforming growth factor‐β3 (TGF‐β3)‐loaded PLGA microparticles (MPs) were mixed with aptamer‐conjugated MECM to simulate anisotropic meniscal regeneration. These three bioactive molecules were delivered sequentially. Apt19S, which exhibited high binding affinity to synovium‐derived MSCs (SMSCs), was quickly released to facilitate the mobilization of ESPCs. CTGF incorporated within PLGA NPs was released rapidly, inducing profibrogenic differentiation, while sustained release of TGF‐β3 in PLGA MPs remodeled the fibrous matrix into fibrocartilaginous matrix. The in vitro results showed that the sequential release of Apt19S/GFs promoted cell migration, proliferation, and fibrocartilaginous differentiation. The in vivo results demonstrated that the sequential release system of Apt/GF‐scaffolds increased neomeniscal formation in rabbit critical‐sized meniscectomies. Thus, the novel delivery system shows potential for guiding meniscal regeneration in situ.

## 1 INTRODUCTION

Meniscus tears are the most prevalent injury of the knee joint and are often accompanied by acute anterior cruciate ligament (ACL) lesions.^1^ Meniscal injuries that occur in the avascular region typically cannot heal and subsequently represent a major osteoarthritis (OA) risk factor.^2^, ^3^ Currently, treatment strategies mainly consist of arthroscopic suture, partial or total meniscectomy, and meniscal allograft transplantation (MAT).^2^ However, arthroscopic meniscectomy inevitably results in progressive cartilage degeneration and OA.^4^ MAT implants have been applied in the clinic, but they are also restricted by several obstacles, such as an insufficient number of donors, implant shrinkage, and the risk of disease transmission.^2^, ^5^ From a clinical perspective, complex meniscus tears are impossible to repair surgically to achieve recovery of the structural and functional properties of the native meniscus. Therefore, the reconstruction of meniscal defects is an enormous challenge for orthopedic surgeons and bioengineering scientists and is of great significance for associated patients.

Recently, in situ meniscal tissue engineering strategies that direct the recruitment and proliferation of endogenous cells and promote their fibrochondrogenic differentiation have attracted tremendous attention.^2^, ^6^ Many researchers have invented functional biomaterials to provide a favorable microenvironment to direct endogenous repair processes without external cell sources, and this cell‐free method has been proven superior to traditional cell‐based therapies and holds promise for clinical translation.^7^, ^8^ Several studies have shown the superior reparative effects of in situ tissue‐engineered meniscal scaffolds on meniscal regeneration. For example, Guo et al. implanted a polycaprolactone (PCL)/meniscus extracellular matrix (MECM) scaffold to repair meniscal defects in rabbits and sheep, and the neomeniscus exhibited similar histological structure, biochemical content, and biomechanical properties to the original.^9^ However, how scaffold materials themselves affect the migration, proliferation, and differentiation of endogenous stem/progenitor cells (ESPCs) remains intricate and hard to manipulate; therefore, these scaffolds are often reinforced by inductive signals to activate the intrinsic regenerative potential of ESPCs. Naturally, the development of cell‐instructive scaffolds that provide various structural, mechanical, and biochemical cues to mobilize and subsequently promote meniscogenic differentiation of ESPCs holds great potential for meniscal regeneration.

The decellularized extracellular matrix of natural tissues has become popular for application in regenerative tissue engineering medicine due to its tissue‐specific chemical components and the microarchitecture of natural extracellular matrices.^4^, ^10^ In a previous study, we reported the potential of meniscal extracellular matrix (MECM) to ameliorate meniscal injuries owing to its excellent microenvironment and ability to dictate the behavior of ESPCs.^9^, ^11^ However, MECM alone exhibits unfavorable gelation performance and poor shape fidelity. Gelatin methacrylate (GelMA), a meniscus‐derived hydrogel, was introduced into our experiment an ideal natural matrix with good biocompatibility and photocrosslinkability.^12^ In addition, ECM‐derived scaffolds are restricted by their poor biomechanical capacity. Thus, we attempted to combine 3D‐printed aligned PCL fibers with MECM‐derived hydrogels to further improve the biomechanical, structural, and functional biomimetics of the meniscal scaffold. Despite its generally known advantages, PCL lacks bioactive cues and has unfavorable hydrophobic properties. To overcome these limitations, surface modification by polydopamine (PDA), which is formed by the oxidation self‐polymerization of dopamine in an alkaline solution, is used to improve the biofunctionality of PCL fibers.^13^

In addition to materials, the delivery of individual bioactive factors or their combinations has been applied in meniscal tissue engineering. For example, Zhang et al. fabricated a dual growth factor (GF)‐functionalized scaffold and placed it in a perfusion bioreactor to acquire consistent dynamic stimuli, ultimately achieving facilitated fibrochondrogenic differentiation. Indeed, in the natural healing process, ESPC migration and participation in regeneration are usually insufficient.^7^ We speculate that the homing and fibrochondrogenic differentiation of ESPCs are sequential events during the process of meniscal regeneration.^4^ Therefore, to recapitulate the inherent staged regeneration cascade, which involves targeted ESPC homing followed by directional differentiation, a sequential drug delivery system was designed and successfully fabricated, and its reparative effects on meniscal defects were examined both in vitro and in vivo.

One of the main challenges for in situ meniscal tissue engineering strategies is the nonspecific and insufficient recruitment of endogenous mesenchymal stem cells (MSCs). Several GFs and chemokines, such as bone morphology protein‐2 and stromal cell‐derived factor‐1, can induce nonspecific migration, including endothelial and inflammatory cells, which can aggravate inflammatory responses and hinder meniscal repair.^1^, ^2^ Aptamers are 20–60 base pair single‐stranded DNA (ssDNA) or RNA fragments that can specifically target numerous small molecules to cells.^14^, ^15^ Aptamers exhibit high specificity and affinity for target ligands without inducing immunogenic or toxic effects. Compared to other targeting agents, such as antibodies, chemokines, GFs, and peptides, aptamers possess greater temperature and pH stability and exhibit a high circulation clearance rate.^16^, ^17^ Recently, researchers have sought to utilize aptamers as therapeutic agents for the recognition and capture of MSCs for tissue engineering applications. A DNA aptamer, Apt19S, was developed to specifically target pluripotent stem cells.^18^ Hu et al. found that when applied in tissue regeneration, Apt19S specifically binds to MSCs, promotes their migration, and subsequently enhances osteochondral regeneration when combined with pro‐chondrogenic molecules.^15^ Therefore, an aptamer‐directed repair system capable of selective recruitment of MSCs to the meniscal defect site would pave a promising new path for in situ knee meniscus repair.

In addition to the deficiency of vascular‐derived stem/progenitor cells in meniscal regeneration, another obstacle is propelling ESPCs, as a heterogeneous population or as single‐cell progenies, into fibrochondrocytes.^19^ Notably, sequentially applied connective tissue growth factor (CTGF) and TGF‐β3 were proven necessary and sufficient to induce MSCs to differentiate into a fibrochondrocyte phenotype in vitro, and when CTGF and TGF‐β3 were incorporated and released from a meniscal scaffold in a spatiotemporally controlled manner, the in vivo results showed functional regeneration and restoration of heterogeneous meniscal tissue phenotypes.^19^ Therefore, we hypothesized that thorough mixing of CTGF and TGF‐β3 and loading onto the integrated scaffold could lead to the differentiation of the migrated MSCs into fibrocartilage. In addition, engineered drug delivery scaffolding systems that not only provide an ECM‐mimicking microenvironment but also control the release of bioactive factors according to biological concerns are beneficial for meniscal regeneration.^4^ Poly lactic*‐co*‐glycolic‐acid (PLGA) is an ideal carrier for GFs for two reasons: excellent biocompatibility and biodegradability, which make it safe, and functional protection of the bioactivity of during encapsulation and delivery.^13^, ^20^ In addition, the small surface area per unit volume of larger‐diameter particles may lead to a reduced drug release rate compared to that of smaller particles.^21^ Therefore, to achieve stepwise differentiation of MSCs, we devised a novel PLGA drug delivery system for the temporally controlled delivery of CTGF and TGF‐β3. A faster release of CTGF was realized by incorporating it into PLGA nanoparticles (NPs), and the sustained release of TGF‐β3 from PLGA microparticles (MPs) was believed to improve the fibrocartilaginous regeneration of meniscal defects.

**FIGURE 1:**
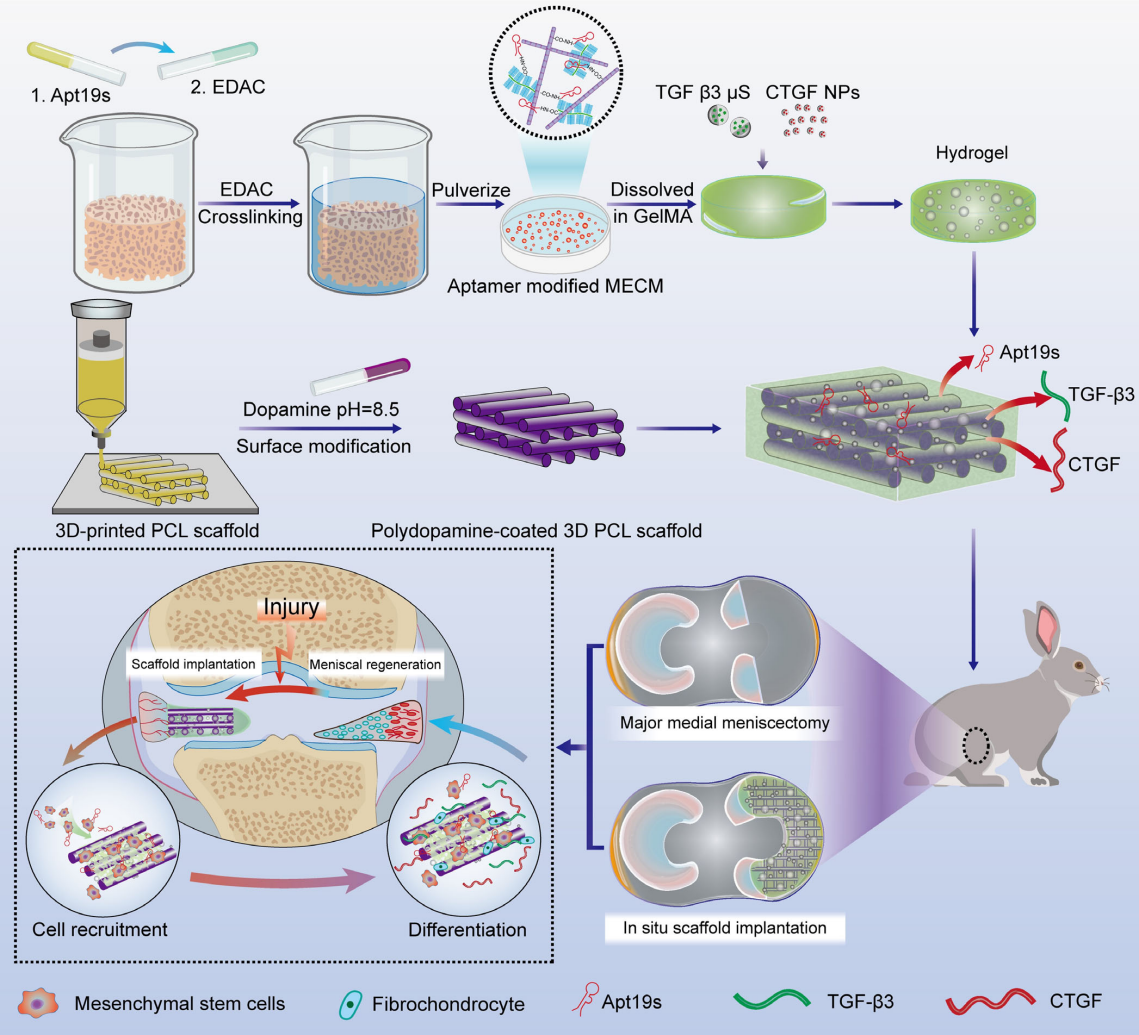
Schematic illustration of the scaffold construction and study design. Aptamer‐functionalized decellularized meniscal extracellular matrix (MECM) combined with transforming growth factor‐β3 (TGF‐β3) microparticles and connective tissue growth factor (CTGF) nanoparticles was first mixed with gelatin methacrylate (GelMA) to form a photocrosslinkable hydrogel (top). Subsequently, the hydrogel was added to the dopamine‐modified 3D‐printed polycaprolactone (PCL) scaffold to develop the functional meniscal scaffold (middle). Finally, the scaffold provides the sustained release of synovium‐derived MSC (SMSC)‐specific chemotactic and differentiative biomolecules and structural support for meniscal regeneration when transplanted into the rabbit model (bottom). Different components are depicted at the bottom of this figure

In this study, an aptamer‐conjugated ECM‐based scaffold was fabricated with the capability to mobilize ESPCs, followed by incorporation with CTGF PLGA NPs and TGF‐β3 MPs to achieve the sequential release of fibrochondrogenic inducers. 3D‐printed PCL fibers were applied as the mechanical support, followed by coating with a PDA layer for its excellent biocompatibility and ability to induce chondrogenesis.^13^, ^22^ As shown in Figure 1, we finally constructed an integrated bioactive scaffold functionalized with Apt19S and GFs, and we hypothesize that this scaffolding system could provide a platform for the sequential release of Apt19S and GFs and efficiently enhance cell homing and differentiation, subsequently achieving cell‐free meniscal regeneration (Figure 1).

## 2 MATERIALS AND METHODS

### 2.1 Scaffold fabrication

#### 2.1.1 Aptamer functionalization of GelMA‐MECM hydrogel

MECM was prepared by physical–chemical methods with some modifications from fresh porcine meniscus tissue as described previously (more details in Data S1 1.1), and the MECM components (such as total collagens, GAGs, and DNA content, etc.) were verified in previous studies by our research team.^23^ For the fabrication of aptamer‐functionalized MECM, a 3% MECM suspension in a cylinder model (3.5 mm in diameter, 20 mm in height) was frozen at −80°C overnight, followed by lyophilization for 24 h to prepare the MECM sponge for the subsequent studies.

An MSC‐specific aptamer named Apt19S was developed according to a previous study,^15^ and the sequences are shown in Table S1. All aptamers were synthesized by Shanghai Sangon Biotech Cooperation in China and purified by HPLC (Agilent, Japan) with a C‐18 column. The binding of amino‐labeled and 5‐carboxyfluorescein (FAM)/amino dual‐labeled aptamers to MECM sponges was performed according to previous studies with some modifications.^15^, ^24^ Briefly, a MECM sponge (3.5 mm in diameter; 1 mm in height) was immersed in 5 ml of morpholinoethanesulfonic acid (MES, 0.1 M, pH = 6) for 30 min at room temperature. The carboxyl groups on the surface of the MECM sponge were activated by adding 20 mg of 1‐ethyl‐3‐[3‐dimethylaminopropyl] carbodiimide hydrochloride (EDC, J&K) and 30 mg of N‐hydroxysuccinimide (NHS, J&K) for 20 min at room temperature. Subsequently, the activation buffer was removed, and the MECM sponge was washed three times with sterile PBS (pH: 7.2). For the conjugation of the aptamer, 10 nmol of modified Apt19S in 5 ml of PBS was added and allowed to react for 12 h in a reciprocating shaker at room temperature. After that, the aptamer‐functionalized MECM sponge was washed three times with PBS to remove residual chemical reagents. Dry GelMA powder was dissolved in sterile PBS to prepare a 20% (w/v) solution, followed by the addition of lithium phenyl‐2,4,6‐trimethylbenzoylphosphinate (LAP). Subsequently, the hydrogel was prepared by mixing isometric ECM and GelMA solutions. Through the aforementioned steps, an aptamer hydrogel consisting of MECM (1.5%, w/v), GelMA (10%, w/v), and LAP (0.25%, w/v) was prepared.

#### 2.1.2 Fabrication of bioactive integrated PCL/hydrogel scaffold

3D‐printing techniques were used to construct PCL scaffolds. In detail, the PCL pellets (Mw 45,000; Sigma, USA) were heated in a printing chamber at 90°C to bring this synthetic polymer to a liquid phase. Then, the melted PCL was extruded through the heated metal nozzle (diameter, 0.25 mm) according to the experimental design by a 3D layer‐by‐layer fused deposition modeling (FDM) printer (FUNMAT, INTAMSYS TECHNOLOGY, China) at 90°C and deposited onto a receiving platform. The 0/90° lay‐down pattern was applied to obtain square pores of scaffolds. Thus, PCL fibers with a diameter of 250 μm and filament gap of 500 μm were produced. Then, the PCL scaffolds were soaked in 2 mg/ml dopamine (Sigma, USA)/Tris–HCl buffer (pH = 8.5), which was stirred gently for 24 h in the dark at room temperature. Next, the scaffolds were removed and rinsed repeatedly with deionized water to remove redundant dopamine and finally allowed to dry naturally. Finally, TGF‐β3 MPs and CTGF NPs were incorporated into MECM‐based hydrogels at a ratio of 20 mg/ml to form GF‐containing hydrogels (more details on TGF‐β3 MPs and CTGF NPs are provided in Data S1 1.2). Finally, the PDA/PCL scaffolds were immersed in liquid MECM‐based hydrogel with or without GFs, placed in an ultrasonicator for another 2 h and subsequently crosslinked under blue light (405 nm) at an intensity of 90 mW/cm^2^ for 5 min for collection.

Based on the above description, the preparation process of the scaffolds is presented in Figure 1. To evaluate the characterization of the functional meniscal scaffolds, simple PCL scaffolds, PCL/PDA scaffolds, PCL/PDA scaffolds with GelMA/MECM (PCL/PDA/GE scaffold), and PCL/PDA/GE scaffolds with blank PLGA MPs/NPs (PCL/PDA/GE‐Blank PLGA scaffold) were fabricated. All scaffolds for in vivo implantation were formed by crescent‐shaped molds (inner diameter: 6 mm; outer diameter: 13 mm).

### 2.2 Scaffold characterization

#### 2.2.1 Macro‐ and microscopic observation

The macroscopic images of the scaffolds were observed under a stereomicroscope (SMZ2; Nikon, Japan). The ultrastructure of the scaffolds was assessed by using a scanning electron microscope (SEM, S‐4800; Hitachi, Japan) after lyophilization for 24 h. The samples were sputter‐coated with Au for 120 s and imaged.

#### 2.2.2 X‐ray photoelectron microscopy

The changes in the surface elements of the scaffolds were studied using X‐ray photoelectron (XPS) spectroscopy (Thermo Scientific ESCALAB 250Xi) with monochromatic Al Kα.

#### 2.2.3 Contact angle

The surface water contact angle was determined by a goniometer (Dataphysics OCA20, Germany) to detect the hydrophilicity of each prepared scaffold. Each group included four samples, and the results were averaged.

#### 2.2.4 Biomechanical assay

The compressive properties of the hydrated scaffold (5 × 5 × 3 mm) and the tensile properties of the hydrated scaffold (4 × 10 × 2 mm) were measured by using a BOSE biomechanical testing machine (BOSE 5100; TE Instruments, New Castle, DE) with a speed of 1 mm/min, the stress–strain curves were recorded until deformation of 80%, and the compression modulus and tensile modulus were calculated from the slope of the curve.

#### 2.2.5 Fourier transform infrared spectroscopy

To determine the presence of specific chemical groups in the integrated scaffolds, samples were analyzed using a Bruker Tensor 27 FTIR spectrometer (Nicolet IS5, Germany) in reflection mode, and all Fourier transform infrared (FTIR) spectra were measured at a resolution of 1 cm^−1^ in the range of 4000–500 cm^−1^.

#### 2.2.6 Dual GF‐release behavior characterization

The release behavior of CTGF (or TGF‐β3) from PLGA NPs (or PLGA MPs) was characterized as follows: briefly, scaffolds containing 40 mg of CTGF NPs (or TGF‐β3 MPs) were immersed in 2.0 ml of PBS working solution with 0.5% (w/v) bovine serum albumin (BSA) and incubated at 37°C. At selected time points (1, 3, 7, 14, 21, 28, 35, and 42 days), 1 ml of supernatant was removed from the tube and replaced with an equal volume of fresh PBS solution. The amount of cumulative released CTGF and TGF‐β3 was measured using a Human TGF‐β3 enzyme‐linked immunosorbent assay (ELISA) (R&D System, USA) kit and a Human CTGF ELISA (MEIMIAN, China) kit, respectively. Finally, the percent GF release curve was plotted. Each sample was assessed in triplicate.

### 2.3 Biocompatibility of the functional meniscal scaffolds

#### 2.3.1 Cell viability and morphology

Synovium‐derived MSCs (SMSCs) and meniscal fibrochondrocytes (MFCs) were isolated from New Zealand white rabbits as described in previous studies,^5^, ^25^ and RAW 264.7 cells (murine macrophage line) were obtained from the American Type Culture Collection (Bethesda, MD) (more details on TGF‐β3 MPs and CTGF NPs are provided in Data S1 1.3). To confirm the viability and morphology of SMSCs on different scaffolds (PCL, PCL/PDA, PCL/PDA/GE, and PCL/PDA/GE‐Blank PLGA scaffold), 5 × 10^5^ SMSCs were seeded and cultured for 4 days before analysis by live/dead staining and 4′,6‐diamidino‐2‐phenylindole (DAPI)/F‐actin staining. Detailed information is given in Data S1 1.4.

#### 2.3.2 Cell proliferation

Cell Counting Kit‐8 (CCK‐8; Dojindo, Japan) was chosen to quantitatively measure the proliferation of SMSCs seeded on different scaffolds (PCL, PCL/PDA, PCL/PDA/GE, and PCL/PDA/GE‐Blank PLGA scaffold) (3 × 10^3^ cells per scaffold, 5 × 5 × 1 mm). After 1, 4, and 7 days of coculture, the culture medium was refreshed with 2 ml of working solution (CCK‐8 reagent/cell culture medium = volume ratio of 1:10) and incubated at 37°C for 2 h. Then, we used a microplate reader (Beckman, Fullerton, CA) to read the absorbance of the test solutions (*n* = 5 per group).

### 2.4 Characterization of functions of aptamer Apt19S

#### 2.4.1 Aptamer binding capacity and specificity assay

Rabbit SMSCs, rabbit MFCs, and mouse macrophages were used to evaluate the binding capacity and specificity of the aptamer. Briefly, 24 h before imaging, 10^5^ cells were seeded onto glass slides. Cells were then washed with PBS and incubated with 10 nM FAM‐labeled aptamer for 8 h at 37°C followed by DAPI staining. After washing three times with PBS, the cells were imaged using a fluorescence microscope. The binding efficiency was determined by the percentage of fluorescent cells in four random fields under high magnification (40×).

#### 2.4.2 Cell migration assay in vitro

A Transwell system (Corning, USA) was used to assess the ability and specificity of the aptamer to recruit SMSCs (Passage 3). Briefly, the SMSC recruitment ability and specificity of aptamers were first determined at different concentrations (1, 10, and 100 nM). In addition, rabbit MFCs and rat macrophage migration assays with 1, 10, and 100 nM aptamer were performed as negative controls. Subsequently, the control group, GE hydrogel group, aptamer‐hydrogel (Apt‐hydrogel) group, and aptamer/GF (Apt/GF)‐hydrogel were tested to verify the recruitment capacity of aptamers conjugated to the GE scaffold. For the SMSC migration experiment procedure, 100 μl of serum‐free DMEM with 2 × 10^4^ resuspended cells was placed in the upper chamber, and 600 μl of DMEM containing 1% FBS (2.5% FBS for rabbit MFCs and macrophages) was placed in the lower chamber with different groups. After 24 h, the cells were fixed with 4% paraformaldehyde for approximately 20 min and stained with crystal violet. Three replicates were carried out per group. The number and species of cells that migrated to the other side of the membrane were calculated by ImageJ.

#### 2.4.3 ESPCs recruitment assay in vivo

Eight healthy, 18‐week‐old male Sprague Dawley rats weighing 200–220 g were employed for the in vivo ESPC recruitment study. This study was designed with four groups as follows: the (A) control group, (B) GE hydrogel group, (C) Apt‐hydrogel group, and (D) Apt/GF‐hydrogel group (*n* = 4 knees in each group). More information about animal surgery is given in Data S1 1.5.

To evaluate the cell lineage recruited to the defect site, CD73 and CD105, which were defined as MSC markers, were assessed by immunofluorescence staining. Briefly, samples (*n* = 4 knees) were first fixed in 4% paraformaldehyde (PFA) for 30 min at room temperature. The target antigens were retrieved by immersing the specimens in 2% sodium citrate for 20 min and blocking them with 10% goat serum. Subsequently, the specimens were incubated overnight at 4°C with CD73 (1:200, Novus Biologicals) and CD105 (1:100, Novus Biologicals). The next day, the samples were washed and incubated with secondary antibodies conjugated with Alexa Fluor488 and Fluor594 (Abcam, Cambridge, UK) for 1 h at room temperature. Next, the samples were counterstained with DAPI (1:1000, Life Technologies) to label the nuclei and observed with a Leica TCS‐SP8 confocal microscope (Leica, Germany). The number of total cells and CD73/CD105 double‐positive cells were analyzed with ImageJ software (USA).

### 2.5 Stepwise fibrocartilaginous differentiation of SMSCs

#### 2.5.1 Fibrocartilage‐related gene expression analysis of different scaffolds in vitro

SMSCs at Passage 3 were seeded on scaffold, Apt‐scaffold, and Apt/GF‐scaffold for in vitro 3D culture to verify the fibrochondrogenic differentiation properties of the three scaffolds. Briefly, scaffold, Apt‐scaffold and Apt/GF‐scaffold were disinfected and placed in 24‐well plates. SMSCs (5 × 10^5^) at Passage 3 were seeded in scaffolds and cultured with GF‐free induction medium (CIM, Cyagen Biosciences, China) consisting of basal medium with chondrogenesis supplementation (dexamethasone, ascorbate, insulin‐transferrinselenium solution, sodium pyruvate, proline). After 7 and 14 days, scaffolds were collected, and total RNA was extracted from samples using commercial TRIzol (Invitrogen, USA) reagent. Reverse transcription to cDNA was then conducted using a ReverTra Ace qPCR RT Kit (FSQ‐201; TOYOBO). A StepOne TM Real‐Time PCR system (Applied Biosystems) with SYBR Green PCR Master Mix (Genestar, USA) was used for the real‐time PCR procedure. The fibrocartilaginous gene primers are listed in Table S2. To obtain credible results, melt curves were assessed following the amplification procedure, and nonspecific amplification was checked. Gene expression was evaluated relative to the housekeeping gene by the ∆Ct method. Four independent assays were performed for each value.

### 2.6 Evaluation of reconstructed meniscus

#### 2.6.1 Surgical procedure

Thirty rabbits were randomized into five groups: (a) the native group (denoted as the sham group), (b) the scaffold group, (c) the Apt‐scaffold group, (d) the Apt/GF‐scaffold group, and (e) the blank group. The method of establishing critical‐size medial meniscectomy was described in our previous study, and more details are described in Data S1 1.6.^25^ After 3 and 6 months, the rabbits were sacrificed, and the meniscus, femurs, and tibial plateaus were sampled for subsequent evaluation.

#### 2.6.2 Magnetic resonance imaging

Magnetic resonance imaging (MRI) images were obtained by using a 7.0 T Bruker Biospec system (Bruker, Ettlingen, Germany). The samples were positioned straight in the MRI‐compatible device for scanning. T2‐weighted imaging (T2WI) was performed (repeated time = 3200 ms/echo time = 65 ms; slice = 15, slice thickness = 1 mm) and further analyzed according to the Whole‐Organ Magnetic Resonance Imaging Score (WORMS) system (Table S3). All of the samples of each group (*n* = 3) were independently scored blindly by three observers.

#### 2.6.3 Macroscopic observations

The degree of the reconstructed meniscus was evaluated via gross examination, and the fully exposed femoral condyle, tibial plateau, and neomeniscus were observed and photographed.

#### 2.6.4 Biochemical contents assays

The COL‐1 and COL‐2 contents of the neomeniscus were assessed by an ELISA kit (Rabbit COL‐1/COL‐2, MEIMIAN, China) following the manufacturer's protocols.

#### 2.6.5 Histological and immunohistochemical analyses

For histological examination, samples were fixed in 4% paraformaldehyde for 2 days, embedded in paraffin, and sectioned into a series of 6 μm slices. The sections were further stained with hematoxylin and eosin (H&E), toluidine blue (TB), and Sirius red (SR) according to the manufacturer's protocols. Additionally, the sections were treated for antigen retrieval and incubated with primary antibodies against collagen I (1:100, Cat. No. NB600‐450; Novus) and collagen II (1:100, Cat. No. NBP2‐33343) overnight at 4°C, followed by incubation with a goat anti‐mouse IgG secondary antibody (1:200; Cat# NB7539; Novus) for 1 h at room temperature. The diaminobenzidine (DAB) substrate system was used for color development.

### 2.7 Evaluation of articular cartilage degradation

For histological analysis, the corresponding femoral condyle and tibial plateau were fixed in 4% paraformaldehyde for 48 h and then decalcified in 20% EDTA (pH 7.2) for 4 weeks. The samples were dehydrated and embedded in paraffin. Seven‐micrometer‐thick serial sections were prepared for staining with Safranin‐O/Fast green (SOG) following the recommended protocol and graded blindly according to the ICRS and Mankin scoring system (Table S4/5).^26^

### 2.8 Statistical analysis

All statistical data are presented as the mean ± standard deviation using SPSS 17.0 statistical software and one‐way or two‐way analysis of variance (multiple groups) to determine the statistical significance. For all analyses, *p* < 0.05 means that the difference is significant.

## 3 RESULTS

### 3.1 Preparation and characterization of the functional meniscal scaffolds

#### 3.1.1 Macro‐ and microstructures of the functional meniscal scaffolds

**FIGURE 2:**
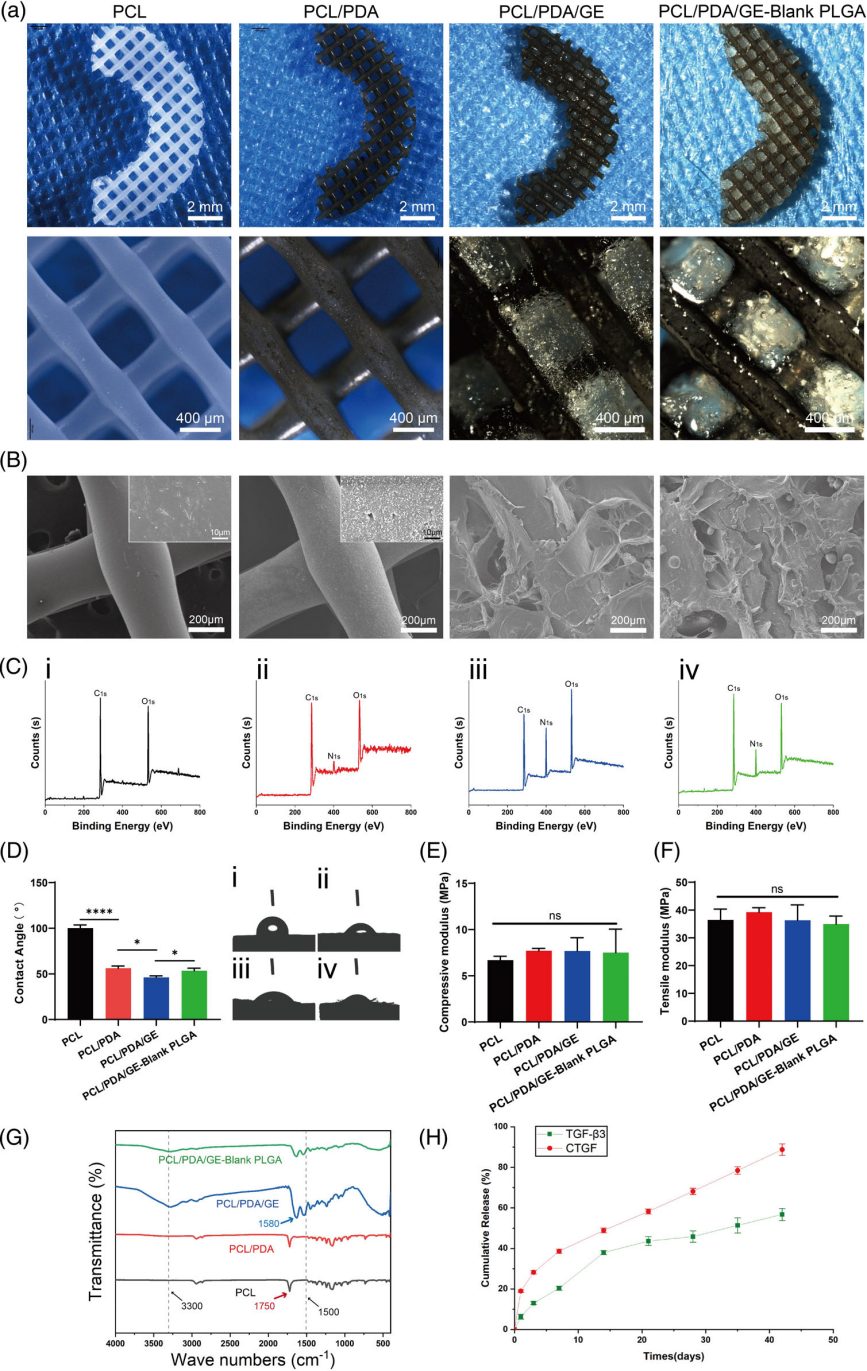
Physicochemical properties of polycaprolactone (PCL), PCL/polydopamine (PDA), PCL/PDA/GE, and PCL/PDA/GE‐blank poly(lactic‐*co*‐glycolic acid) (PLGA) scaffolds. (a) Macroscopic observation and (b) SEM images of four different scaffolds. (c) Surface elemental compositions of four different scaffolds determined by X‐ray photoelectron microscopy (XPS) analysis. (d) Contact angle quantitative evaluation of different scaffolds (*n* = 4, i, represents the PCL scaffold; ii, represents the PCL/PDA scaffold; iii, represents the PCL/PDA/GE scaffold; and iv, represents the PCL/PDA/GE‐blank PLGA scaffold). Compressive modulus (e) and tensile modulus (f) properties of different scaffolds. (g) Fourier transform infrared (FTIR) spectra of different scaffolds. (h) Cumulative release profiles of connective tissue growth factor (CTGF) and transforming growth factor‐β3 (TGF‐β3) (*n* = 3). Data are means ± SD. One‐way analysis of variance in d–f. ns indicates no significant difference, *p < 0.05, **p < 0.01, ***p < 0.005, ****p < 0.001

Macro‐ and microstructure observation images are shown in Figure 2a,b. After surface modification by PDA, the surface of the scaffold became black. The structural geometry studies by stereomicroscopy and SEM analysis exhibited heterogeneous distributions of macro‐ and micropores. The macropores of the four scaffolds were in the range of 500–600 μm, while the micropores of the PCL/PDA/GE scaffold and PCL/PDA/GE‐blank PLGA scaffold were mainly in the range of 60–220 μm (Figure S1) and 60–140 μm (Figure S2), respectively, which favored cell adhesion, retention, and differentiation. We also found that the PLGA MPs were scattered and distributed on the scaffold, and the microstructures of the PLGA NPs and MPs are shown in Figures S3 and 4.

#### 3.1.2 X‐ray photoelectron microscopy

XPS spectra were applied to evaluate the chemical composition of the four as‐prepared scaffolds. The results showed that only the elements C and O were detected in the bare PCL fiber sample (Figure S5). After PDA surface modification, nitrogen (N) appeared (the N1s spectra corresponding to the NH‐bonded to the carbon chain in the PDA), which indicates the successful introduction of amino groups onto the PCL fibers (Figure 2c). The PCL/PDA scaffold exhibited increased N content (5.94%) compared to the PCL scaffold, indicative of successful PDA mobilization (Figure 2c‐i/ii, Table S6). In addition, with the incorporation of GE hydrogel, the N content of the PCL/PDA/GE (17.84%) scaffold was dramatically higher than that of the PCL/PDA scaffold (Figure 2c‐ii/iii, Table S6). However, the addition of PLGA into PCL/PDA/GE decreased its N content (10.26%) due to the relative dilution of the N‐containing components (Figure 2c‐iii/iv, Table S6).

#### 3.1.3 Contact angle evaluation

Because increased surface hydrophilicity of the PCL scaffold was conducive to cell adhesion and hybrid scaffold construction, surface modification by PDA, which is formed by the oxidation self‐polymerization of dopamine in an alkaline solution, was used to improve the biofunctionality of PCL fibers. The contact angle of the PCL was 100.01 ± 3.452°. After modification, the contact angles of the other three groups (PCL/PDA, PCL/PDA/GE, PCL/PDA/GE‐Blank PLGA) were 56.23 ± 2.398°, 46.13 ± 1.670°, and 53.40 ± 2.800°, respectively, all of which were significantly lower than that of the PCL group (Figure 2d), showing great improvement in the hydrophilicity to promote cell attachment.

#### 3.1.4 Biomechanical assay

In the bioprinting of biomimetic scaffolds, unreasonable model and parameter design commonly lead to unrepeatable and unstable results. Therefore, we investigated the biomimetic compressive and tensile mechanical properties of the 3D‐printed scaffold in Figure 2e,f. The compressive and tensile moduli were calculated based on the slope of the linear region of the stress–strain curve. The compressive moduli of the four scaffolds were 6.682 ± 0.4179 , 7.701 ± 0.2513, 7.678 ± 1.429, and 7.506 ± 2.537 MPa, respectively, and the tensile moduli was 36.45 ± 3.874, 39.21 ± 1.641, 36.29 ± 5.552, and 34.96 ± 2.852 MPa, which showed desirable mechanical performance compared to the native meniscus (compressive modulus: 3–6 MPa; tensile modulus: 16–45 MPa).^25^, ^27^, ^28^ There were no significant differences between these scaffolds.

#### 3.1.5 FTIR spectroscopy

The structural changes in the materials were characterized by using FTIR spectroscopy. As depicted in Figure 2g, the characteristic peak at 1750 cm^−1^ represents the carbonyl group (C═O) of PCL. After surface modification by PDA, the signals of PCL/PDA that appeared at 1500 cm^−1^ were assigned to the bending vibrations of the secondary amine bond, and the peak intensities at 1600 cm^−1^ represented the bending vibrations of the primary amine bond. Moreover, the introduction of GelMA and MECM, which contained many hydroxyl groups and amine groups (N—H bend), resulted in wide protrusions at approximately 3300 cm^−1^ and 1580 cm^−1^, respectively. However, the hydroxyl region of the PCL/PDA/GE‐blank PLGA spectrum was not obvious, possibly because the addition of PLGA decreased the relative proportion of this chemical group. The FTIR spectra of the three as‐prepared samples showed some significant differences that proved the successful surface modification and fabrication of the hybrid scaffold.

#### 3.1.6 Dual GF‐release behavior characterization

The release profiles of TGF‐β3 and CTGF from the 3D‐printed scaffolds in vitro were investigated using the corresponding ELISA kits, and the results are presented in Figure 2h. There was a burst release of nearly 20% of the total CTGF on the first day; the release subsequently slowed, with approximately 30% of the total CTGF released after 7 days. In contrast, the overall release of TGF‐β3 from the scaffold was comparatively slow and gentle. An initial burst release of 8% of the total TGF‐β3 was observed on the first day; at 14 days, nearly 40% of the TGF‐β3 was released from the scaffold, and the remaining percentage of CTGF was also more than 50%. Moreover, both TGF‐β3 and CTGF were released for as long as 6 weeks, indicating the sequential release behavior of the two bioactive molecules from the 3D‐printed scaffold. These results revealed that the microsphere/hydrogel composite drug delivery system protected and sequentially presented the bioactivity of both GFs, which improved the ability of the scaffold to induce ESPC differentiation.

### 3.2 Biocompatibility of the functional meniscal scaffolds

**FIGURE 3:**
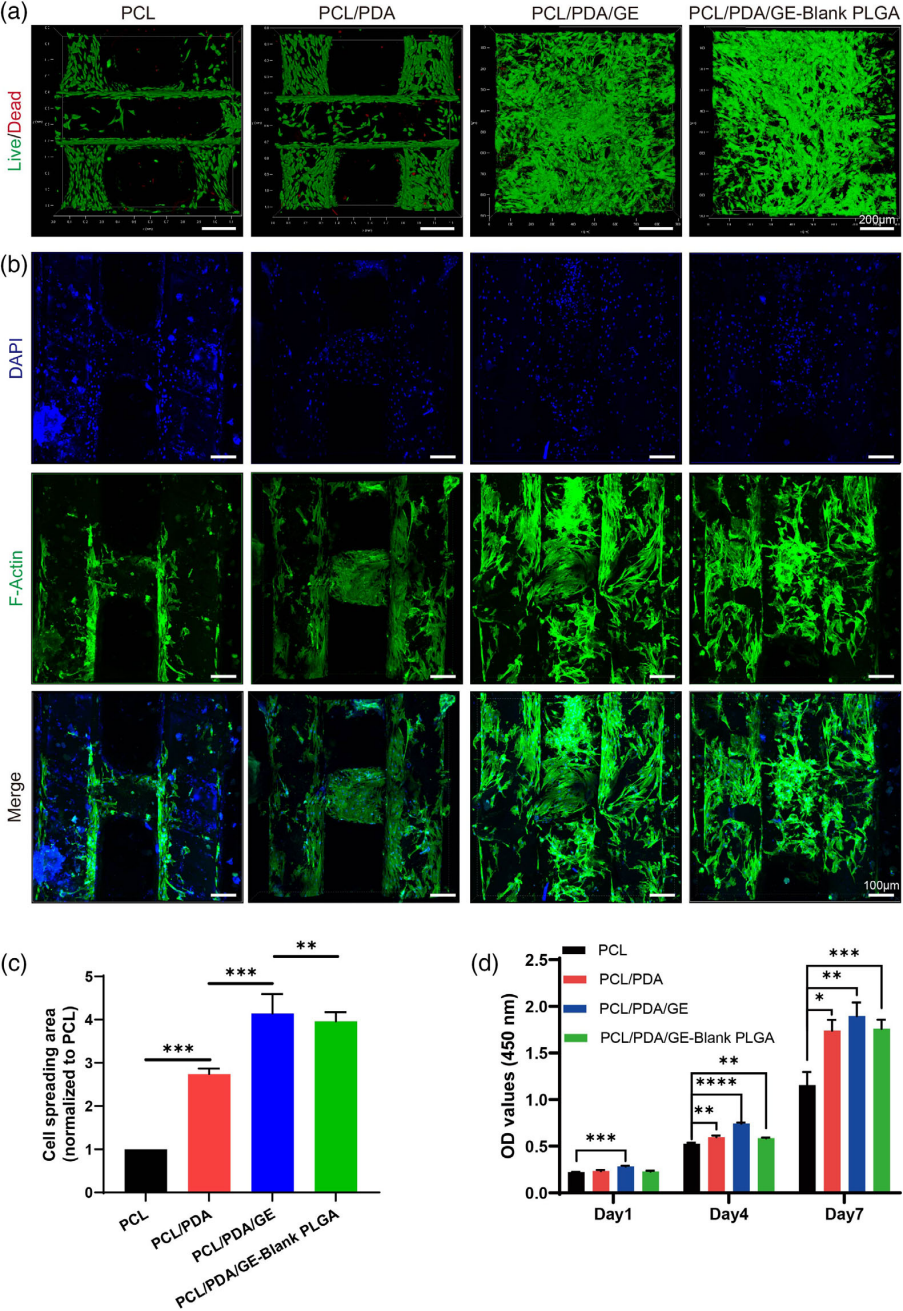
Cytocompatibility of polycaprolactone (PCL), PCL/PDA, PCL/polydopamine (PDA)/GE, and PCL/PDA/GE‐blank PLGA scaffolds. (a) Live/dead staining of different scaffolds. (b) DAPI (blue) and F‐actin staining (green) of cells cultured on different scaffolds for Day 7. (c) Quantitative analysis of the cell spreading area (*n* = 3, normalized to the PCL group). (d) CCK‐8 evaluation of cells cultured on different scaffolds for Days 1, 4, and 7 (*n* = 5). Data are means ± SD. One‐way analysis of variance in (c) and two‐way analysis of variance in (d). *p < 0.05, **p < 0.01, ***p < 0.005, ****p < 0.001

The biocompatibility (such as cell viability, cytoskeleton, and proliferation) of different scaffolds was determined by live/dead staining, DAPI/F‐actin staining, and the CCK‐8 assay, as shown in Figure 3. First, confocal microscopy of the four groups showed almost all live cells (green color) without any obvious dead cells (red color) in all of the scaffolds, indicating that the microenvironment of each scaffold was beneficial to cell growth (Figure 3a). Subsequently, we explored the cell morphology of all groups by DAPI/F‐actin staining, and each group exhibited different stretching characteristics (Figure 3b). The PCL/PDA group showed better ductility than the PCL group, whereas the PCL/PDA/GE and PCL/PDA/GE‐blank PLGA groups showed the best cell spreading among the four groups. To further evaluate the cell morphology of different scaffolds, the cell spreading area normalized to the PCL group was quantitatively depicted in Figure 3c, and the results showed that the PDA‐modified PCL scaffold enhanced cell stretching compared to the pure PCL scaffold. Although the introduction of blank PLGA MPs might reduce the cell spreading area to some extent, the PCL/PDA/GE scaffold showed the largest cell area among the four groups, which was consistent with the confocal images of Figure 3b. Furthermore, the CCK‐8 assay determined that the number of SMSCs cultured onto the scaffold gradually increased over time from Day 1 to Day 7, and the cell proliferation rate of the PCL/PDA scaffold was higher than that of the PCL scaffold. The growth rate of the PCL/PDA/GE group was the highest among the four groups (Figure 3d). Collectively, these results indicated that the PCL/PDA/GE and PCL/PDA/GE‐blank PLGA scaffolds exhibited good biocompatibility and could provide an optimal platform for the adhesion, proliferation, and spreading of migrated cells.

### 3.3 Apt19S promoted the specific migration of MSCs in vitro and in vivo

**FIGURE 4:**
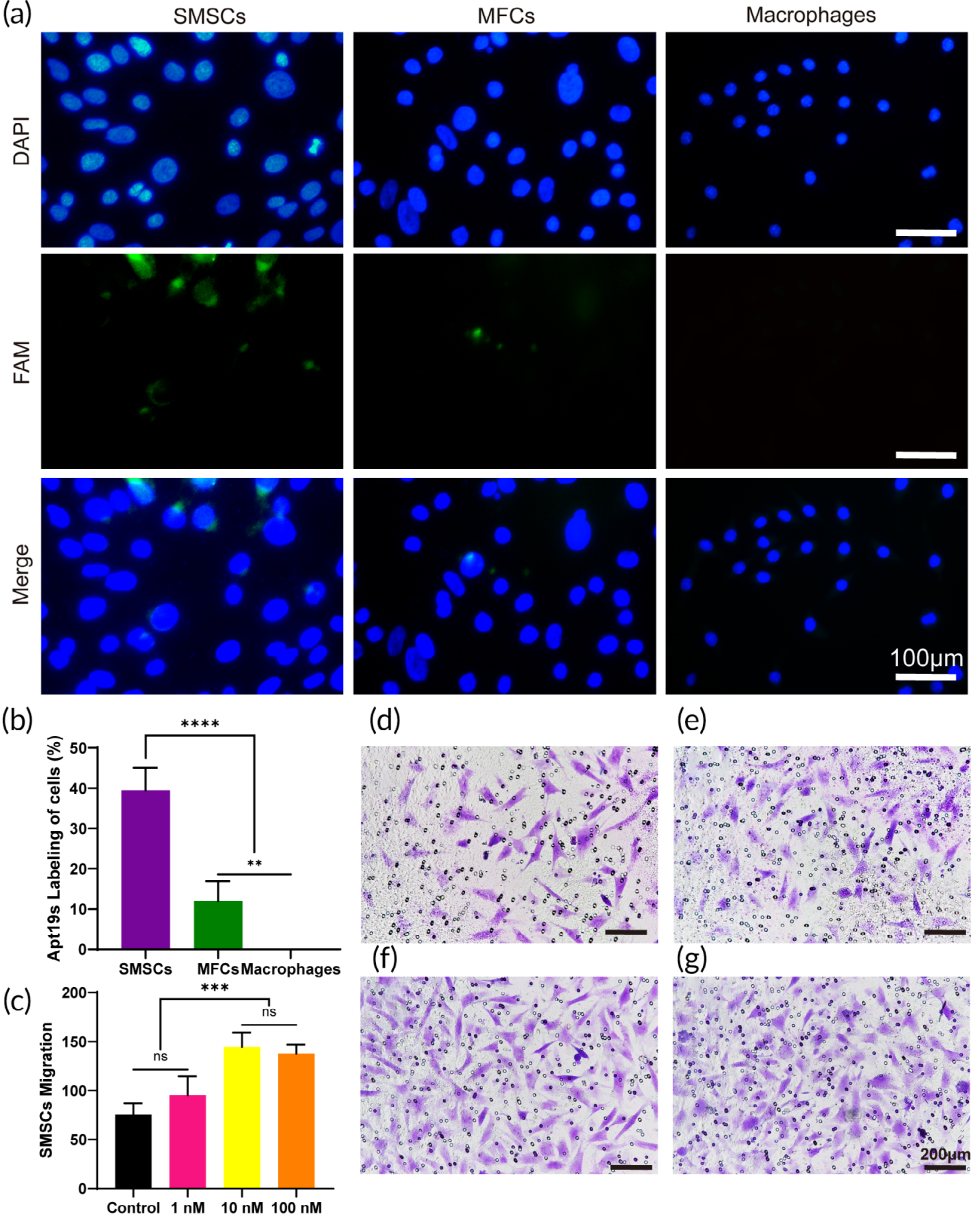
Specific binding and recruitment ability of Apt19S on synovium‐derived MSCs (SMSCs). (a) Confocal immunofluorescence images (a) and quantitative analyses (b) of FAM‐labeled Apt19S in rabbit SMSCs, rabbit meniscal fibrochondrocytes (MFCs), and mouse macrophages (*n* = 4). (c) Statistical analysis and crystal staining (d–g) of SMSC migration toward the (d) control group, (e) 1 nM Apt19s, (f) 10 nM Apt19s, and (g) 100 nM Apt19S in a Transwell system (*n* = 3). Data are means ± SD. One‐way analysis of variance in (b) and (c). ns indicates no significant difference, *p < 0.05, **p < 0.01, ***p < 0.005, ****p < 0.001

To investigate whether Apt19S could specifically recognize and bind SMSCs, the FAM‐labeled aptamer was incubated with different cells, including rabbit SMSCs, rabbit MFCs, and mouse macrophages, and detected by fluorescence microscopy. When FAM‐labeled Apt19S was incubated with SMSCs, obvious overlap of the green fluorescence representing the Apt19S and SMSC surfaces was observed (Figure 4a). However, when FAM‐labeled Apt19S was incubated with rabbit MFCs and mouse macrophages, only scattered green fluorescence was observed on the surface of MFCs, and no green fluorescence was observed in the macrophage group. In addition, the quantitative analysis of aptamer‐labeled cells showed that nearly 40% of SMSCs were labeled by green fluorescence, which was clearly higher than the percentage of other cells labeled, confirming the specific recognition and binding capacity of Apt19S to rabbit SMSCs (Figure 4b).

**FIGURE 5:**
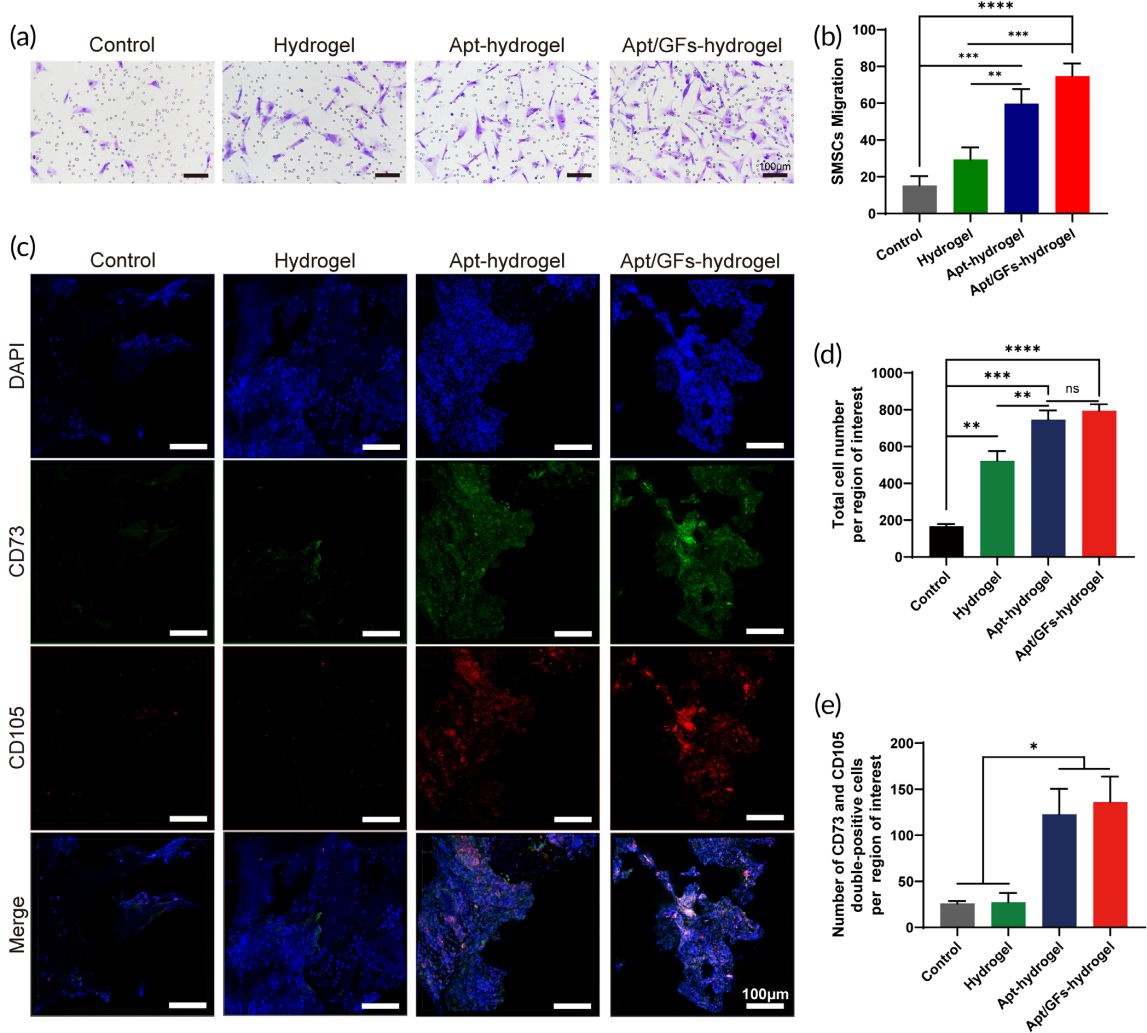
In vitro and in vivo recruitment of mesenchymal stem cells (MSCs) by different groups. (a) Crystal violet staining of the migrated synovium‐derived MSCs (SMSCs) in the control group, hydrogel group, Apt‐hydrogel group, and Apt/GF‐hydrogel group. (b) Statistical analysis of migrated SMSCs in different scaffolds (*n* = 3). (c) Confocal images of endogenous cell recruitment in different groups. (d) Total number of cells migrating into the meniscal defect site (*n* = 4). (e) Number of CD73/CD105 double‐positive cells recruited into the meniscal defect site (*n* = 4). Data are means ± SD. One‐way analysis of variance in (b), (d), and (e). ns indicates no significant difference, *p < 0.05, **p < 0.01, ***p < 0.005, ****p < 0.001

It has been acknowledged that sufficient and specific ESPCs are indispensable for meniscal repair because a lack of MSCs might induce poor healing.^29^, ^30^ Therefore, we explored the effects of Apt19S on MSC‐specific recruitment and mobilization ability in vitro and in vivo. Transwell assays were conducted to investigate in vitro SMSC migration toward different concentrations of Apt19S, and the results in Figure 4c–g show that the numbers of SMSCs migrating to 10 and 100 nM Apt19S were significantly higher than those in the 1 nM Apt19S and control groups. Additionally, the numbers of migrated SMSCs showed no significant difference in the 10 and 100 nM Apt19S groups, indicating that 10 nM Apt19S might be sufficient for recruiting SMSCs. Moreover, we explored whether Apt19S could recruit other cell types, including MFCs and macrophages, which might participate in the repair process. We can see from the results in Figures S6 and 7 that there was no significant difference in the numbers of MFCs and macrophages in the control group and at different concentrations of aptamers. We further assessed the effects of Apt19S‐conjugated hydrogels on MSC mobilization in vitro. The Apt19S‐containing hydrogel was placed below Transwell inserts, and histological staining by crystal violet revealed a uniform distribution of migrated cells. After being cultured for 24 h, the numbers of migrated SMSCs in the Apt‐hydrogel and Apt/GF‐hydrogel groups were significantly greater than those in the control group and hydrogel group, and there was no obvious difference between the two groups. (Figure 5a,b). In summary, the aforementioned results showed that Apt19S could specifically recognize, bind, and capture SMSCs rather than MFCs and macrophages and could enhance MSC recruitment and mobilization when released from the hydrogel.

Although the in vitro results demonstrated that Apt19S could specifically recognize and recruit SMSCs, its in vivo MSC recruitment ability remained unknown. Therefore, in vivo migration was assessed by implanting an Apt‐hydrogel in the rat meniscus and harvesting 1 week post surgery. Confocal imaging showed that the total cell numbers in the meniscus defects of the three hydrogel groups were greater than those in the control group (Figure 5c,d). Total cell numbers of the Apt‐hydrogel group were higher than that of hydrogel group, while no significant difference from the Apt/GF‐hydrogel group was observed, demonstrating that Apt19S rather than GFs could promote cell migration (Figure 5c,d). Importantly, we chose CD73 and CD105 dual MSC‐specific markers to identify ESPCs that migrated to defects, and the results showed that the number of CD73/CD105 double‐positive cells in the Apt‐hydrogel and Apt/GF‐hydrogel groups was dramatically higher than that in the control and hydrogel groups (Figure 5c,e). Therefore, the in vivo results indicated that Apt19S could specifically capture ESPCs and promote their migration to meniscal defect sites during the regeneration process.

### 3.4 Apt/GF‐scaffolds enhanced in vitro stepwise fibrocartilaginous differentiation

**FIGURE 6:**
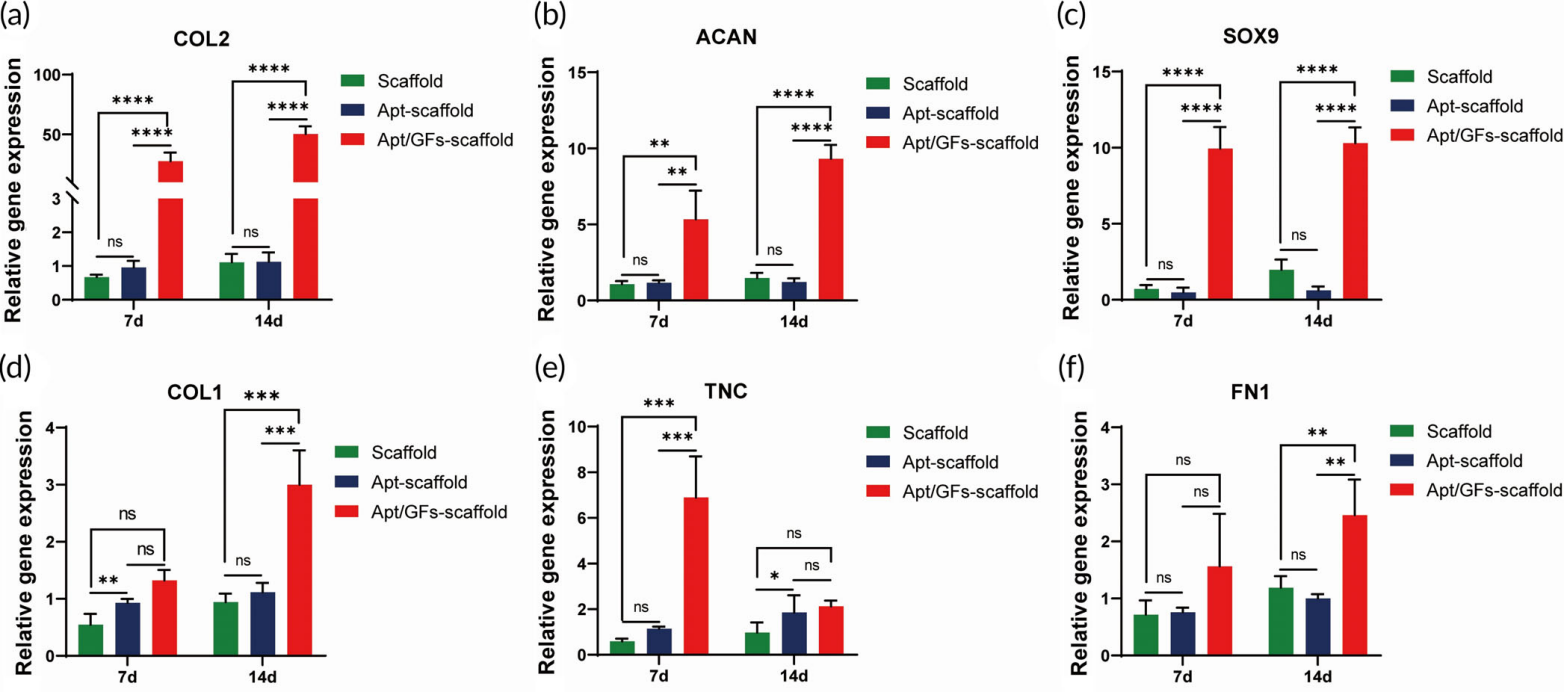
In vitro chondrogenic and fibrochondrogenic gene expression profiles of synovium‐derived MSCs (SMSCs) cultured in scaffolds, Apt‐scaffolds and apt/GF‐scaffolds for 7 and 14 days (*n* = 4). Data are means ± SD. Two‐way analysis of variance in (a)–(f). ns, indicates no significant differences, *p < 0.05, **p < 0.01, ***p < 0.005, ****p < 0.001

In addition to confirming the good biocompatibility and specific recruitment ability if the scaffolds, we investigated the in vitro expression of meniscus‐related genes in different scaffolds through RT‐PCR assays. Chondrogenic genes (such as COL2, ACAN, and SOX9) and fibrochondrogenic genes (COL1, TNC, and FN1) in different groups were detected and are shown in Figure 6. Although the gene expression levels of COL1 (7 days, Figure 6d) and TNC (14 days, Figure 6e) in the Apt‐scaffold group were higher than those in the scaffold group, no obvious difference in the chondrogenic genes COL2, ACAN, and SOX9 or the fibrochondrogenic gene FN1 expression appeared in the scaffold‐ and aptamer‐functionalized scaffold after 7 and 14 days of coculture of SMSCs with the scaffolds. With the incorporation of CTGF NPs and TGF‐β3 MPs, a significant increase in both chondrogenic and fibrochondrogenic differentiation was observed at 7 and 14 days. In addition, we discovered that the chondrogenic gene expression of COL2 and ACAN in the Apt/GF‐scaffold group exhibited an increasing tendency over time from 7 to 14 days, whereas the other groups showed no such tendency, indicating that GFs might play an important role in promoting meniscus‐related gene expression (Figure 6a–c). The TNC expression of the Apt/GF‐scaffolds exhibited a decreasing tendency after 7 and 14 days of culture, which was opposite to the expression patterns of the other two fibrochondrogenic genes in Apt/GF‐scaffolds (Figure 6d,f). These results indicated that the combination of CTGF and TGF‐β3, which were sequentially released from fabricated scaffolds, could significantly stimulate the fibrochondrogenic and chondrogenic differentiation of SMSCs in vitro.

### 3.5 In vivo meniscal repair studies

#### 3.5.1 MRI evaluation

**FIGURE 7:**
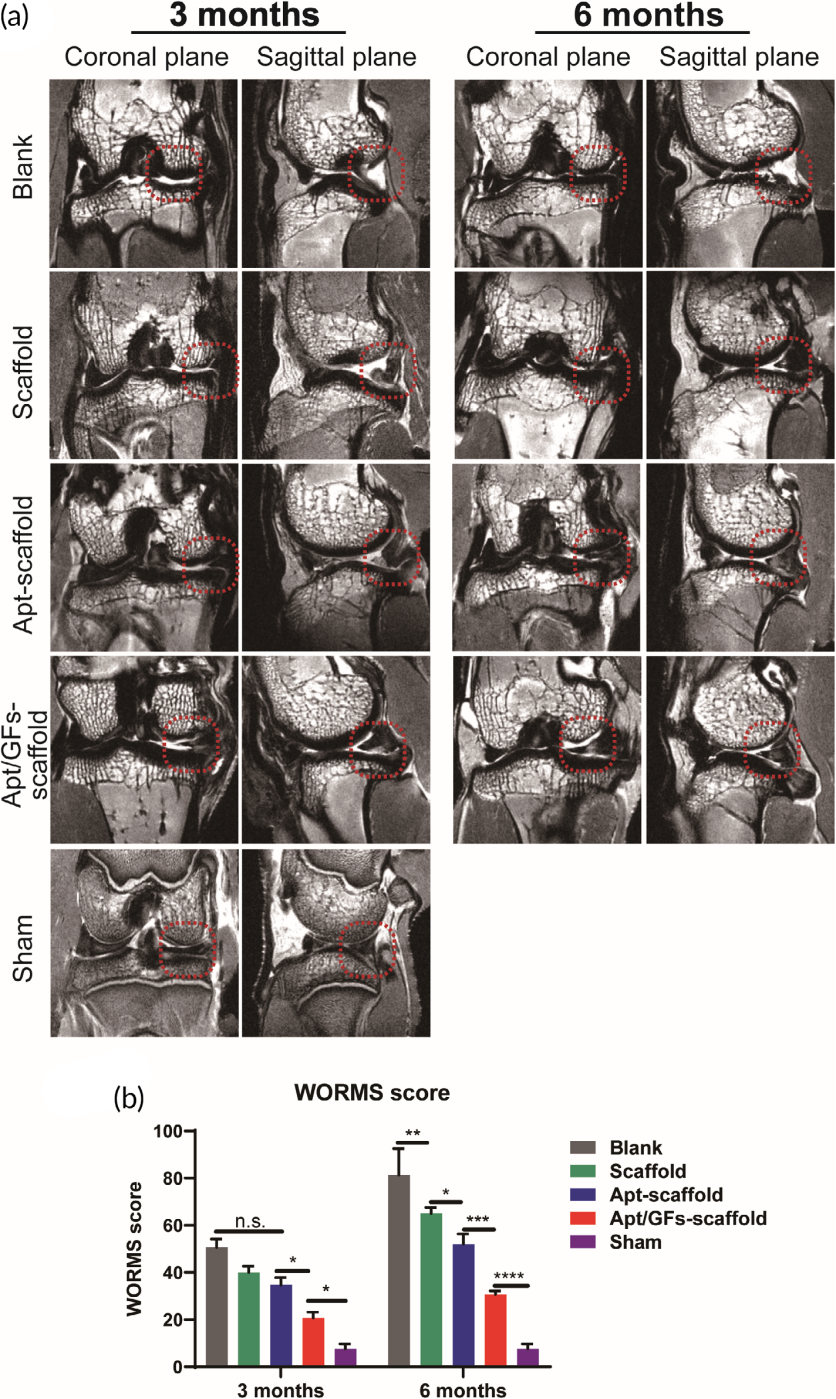
Imagological evaluation. Magnetic resonance imaging (MRI) (a) and Whole‐Organ Magnetic Resonance Imaging Score (WORMS) assessment (b) of rabbit knees (*n* = 3). Data are means ± SD. Two‐way analysis of variance in (b). ns indicates no significant difference, *p < 0.05, **p < 0.01, ***p < 0.005, ****p < 0.001

MRI analysis was conducted to compare regenerative meniscal tissue in defect sites within different groups. Two‐dimensional images were captured to show the tissue morphogenesis in both coronal and sagittal planes. The regenerated meniscus and articular cartilage were marked by a red circle (Figure 7a). Generally, inflammatory signals were identified in the blank group, whereas mild inflammatory signals were identified in the Apt/GF‐scaffold group, and moderate inflammatory signals were identified in the scaffold group and the Apt‐scaffold group. At 3 months, the defect in the blank group remained, and no obvious meniscus‐like tissue was observed in the triangular region of the meniscus, while only a small amount of neotissue formation was observed at 6 months and the articular cartilage was severely damaged. In the Apt/GF‐scaffold group, the regenerated meniscus was observed at 3 months postimplantation and grew over time. Moreover, the cartilage surface was smooth. Neomeniscus could also be observed in the Apt‐scaffold group, but the size of the meniscus tissue was smaller than that in the Apt/GF‐scaffold group at both time points. The cartilage surface in the Apt‐scaffold group was not as smooth as that in the Apt/GF‐scaffold group. For the scaffold group, the image exhibited a minor meniscus profile with unfavorable density and integrity, and the signals were not clear. Surface cartilage damage could also be observed. The WORMS score for the Apt/GF‐scaffold group was significantly lower (better) than that for the scaffold group and the Apt‐scaffold group and was closest to that of the sham group (Figure 7b), which indicates that this integrated bioactive scaffold has excellent regeneration effects.

#### 3.5.2 Macroscopic and biochemical evaluation

**FIGURE 8:**
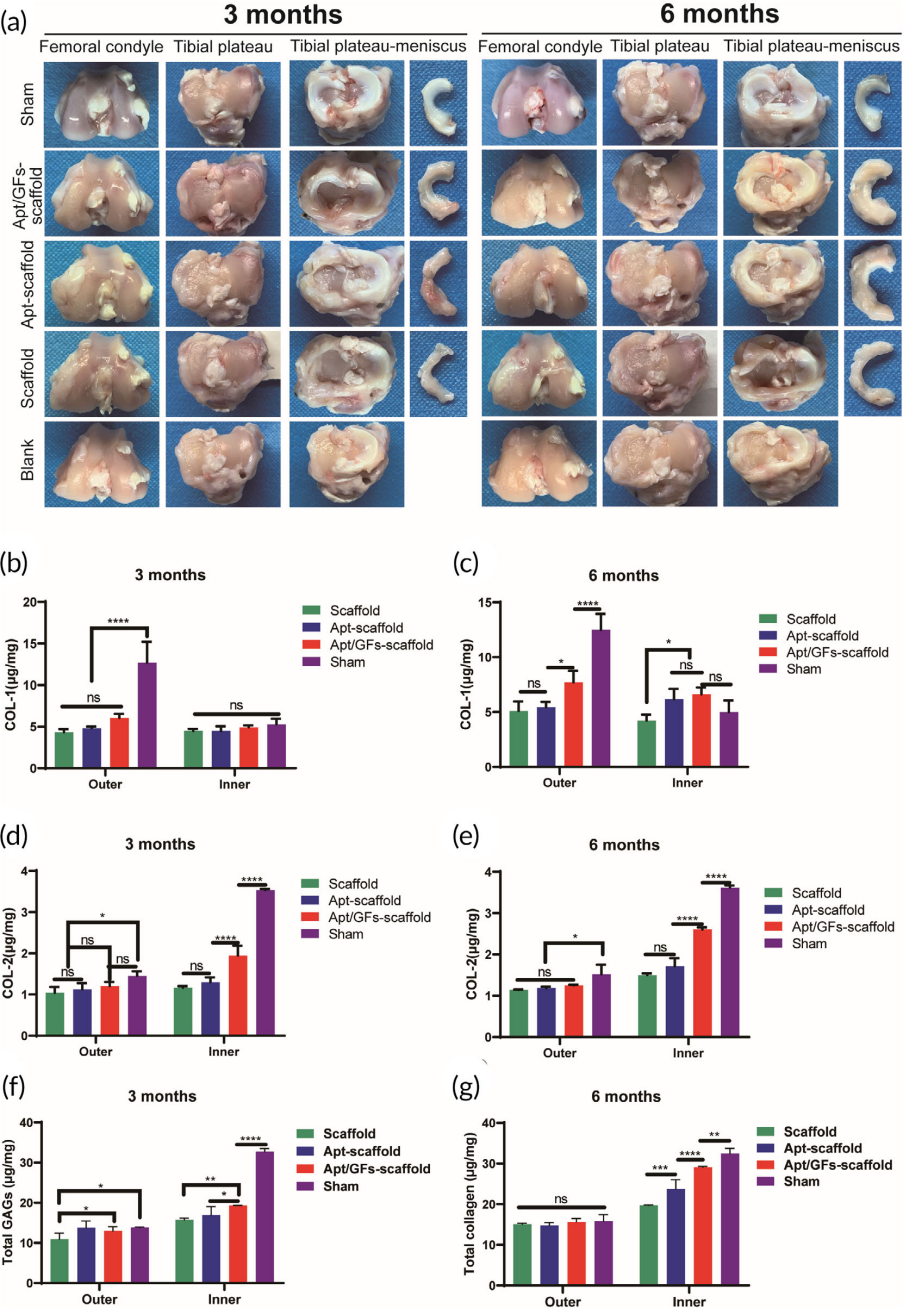
Macroscopic and biochemical assessment of regenerated meniscus. (a) Macroscopic observation of the neomeniscus and the femoral condyles and tibial plateaus. COL‐1 quantitative detection of the regenerated meniscus at 3 months (b) and 6 months (c) postsurgery (*n* = 4). COL‐2 quantitative detection of the regenerated meniscus at 3 months (d) and 6 months (e) postsurgery (*n* = 4). Total GAG quantitative detection of the regenerated meniscus at 3 months (f) and 6 months (g) postsurgery (*n* = 4). Data are means ± SD. Two‐way analysis of variance in (b–g). ns, no significant difference, *p < 0.05, **p < 0.01, ***p < 0.005, ****p < 0.001

The macroscopic morphologies of the regenerated menisci and the corresponding femoral condyles, as well as the tibial plateaus, were observed after knee joint dissection (Figure 8a). For the macroscopic evaluation of meniscal tissue, we found that no obvious regenerated meniscus but a small amount of synovial hyperplasia could be observed in the blank group at both time points. After 3 months of implantation, all three scaffold groups (the scaffold group, the Apt‐scaffold group, and the Apt/GF‐scaffold group) formed meniscus‐like tissue in situ. In the Apt‐scaffold group and the Apt/GF‐scaffold group, the regenerated menisci were fully intact and presented a natural appearance with a shiny white color compared to the menisci in the scaffold group and were better integrated into the joint. However, the gross appearance of the neomeniscus in the Apt/GF‐scaffold group was superior to that in the Apt‐scaffold group at the 3‐ and 6‐month time points, resembling that in the sham group. Moreover, in the Apt/GF‐scaffold group, the neomeniscus had a more complete and uniform shape and better integration with the joint at both time points than those in the scaffold group and the Apt‐scaffold group. In general, better meniscal tissue formation was observed at 6 months in all of the scaffold groups.

In addition, COL‐1, COL‐2, and total GAG quantitative detection of the inner and outer regions of the meniscus at 3 months and 6 months was used to evaluate the biochemical content. At 3 months, in the Apt/GF‐scaffold group, a higher COL‐1 content in the outer region was detected than in the other two scaffold groups, whereas higher COL‐2 and GAG contents were detected in the inner region, and the biochemical content of the inner and outer regions was similar to that of the native meniscus (Figure 8b,d,f). At 6 months, the biochemical content of each group was increased, but the trend was consistent at 3 months, especially in the Apt/GF‐scaffold group, where the ECM organization resembled that of the native meniscus (Figure 8c,e,g).

#### 3.5.3 Histological and immunohistochemical analyses of the repaired meniscus

**FIGURE 9:**
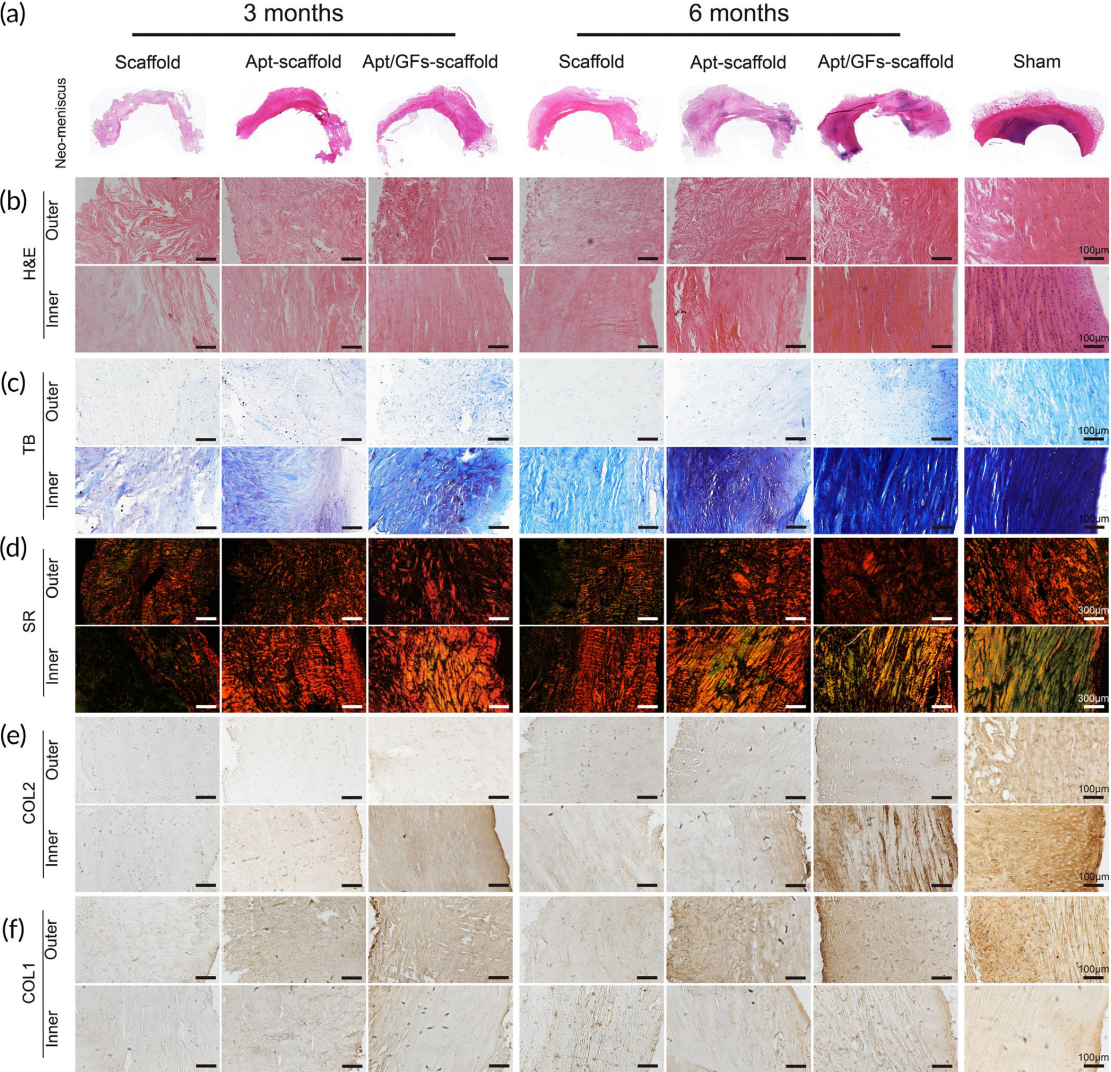
Histological and immunohistochemical staining of repaired meniscus and cartilage of the femoral condyle and tibial plateau. (a) General view of regenerated meniscus stained by H&E, (b) H&E staining, (c) TB staining, (d) SR staining, (e) COL2, and (f) COL1 immunohistochemical images for regenerated meniscus

To examine the histological features of implants, H&E staining, TB staining, SR staining, and immunohistochemical staining were performed. In all groups, no obvious evidence of neutrophil infiltration was observed. In the Apt/GF‐scaffold group, numerous elongated fibroblast‐like cells or round chondrocyte‐like cells could be observed by H&E staining at both time points (Figure 9a,b). The main differences observed between the two time points were more chondrocyte‐like cells and fewer fibroblast‐like cells in the inner region of the neomeniscus at 6 months than at 3 months. TB and immunohistochemical staining for collagen II in the inner region and immunohistochemical staining for collagen I in the outer region were strong, and the staining intensity was stronger at 6 months than at 3 months (Figure 9c,e,f), revealing similar cell morphology to that of the native meniscus in the sham group. In SR staining (Figure 9d), red and birefringent fibers represented collagen I, and collagen III was green with weak birefringence. Generally, the collagen fibers were better ordered at 6 months than at 3 months and were nearly continuous in all groups. In the Apt/GF‐scaffold group, more ordered and tightly arranged collagen fibers were observed in both the outer and inner regions, resembling those in the native group. In the Apt‐scaffold group, only several elongated fibroblast‐like cells were detected, and TB staining and collagen I and collagen II immunohistochemical staining were weakly positive at 3 months postimplantation. In addition, SR staining revealed a tangled arrangement of collagen fibers. However, the cell numbers increased obviously over time. In addition, we found more fibroblast‐like cells and some chondrocyte‐like cells in the inner region of the neomenisci, and at 6 months after surgery, staining for TB, collagen I, and collagen II became positive, and SR staining showed a slightly more ordered arrangement of collagen fibers than before in the Apt‐scaffold group. The staining intensity of the regenerated meniscus in the Apt‐scaffold group was weaker than that in the Apt/GF‐scaffold group but stronger than that in the scaffold group at each time point. In the scaffold group, there were a small number of cells at 3 months postsurgery, and the cell numbers increased slightly by 6 months after implantation. TB staining and collagen I and collagen II immunohistochemical staining were very weak at each time point. In addition, SR staining revealed the most tangled and loose arrangement of collagen fibers compared to the other groups.

#### 3.5.4 Apt/GF‐scaffolds achieved better protective effects on articular cartilage

**FIGURE 10:**
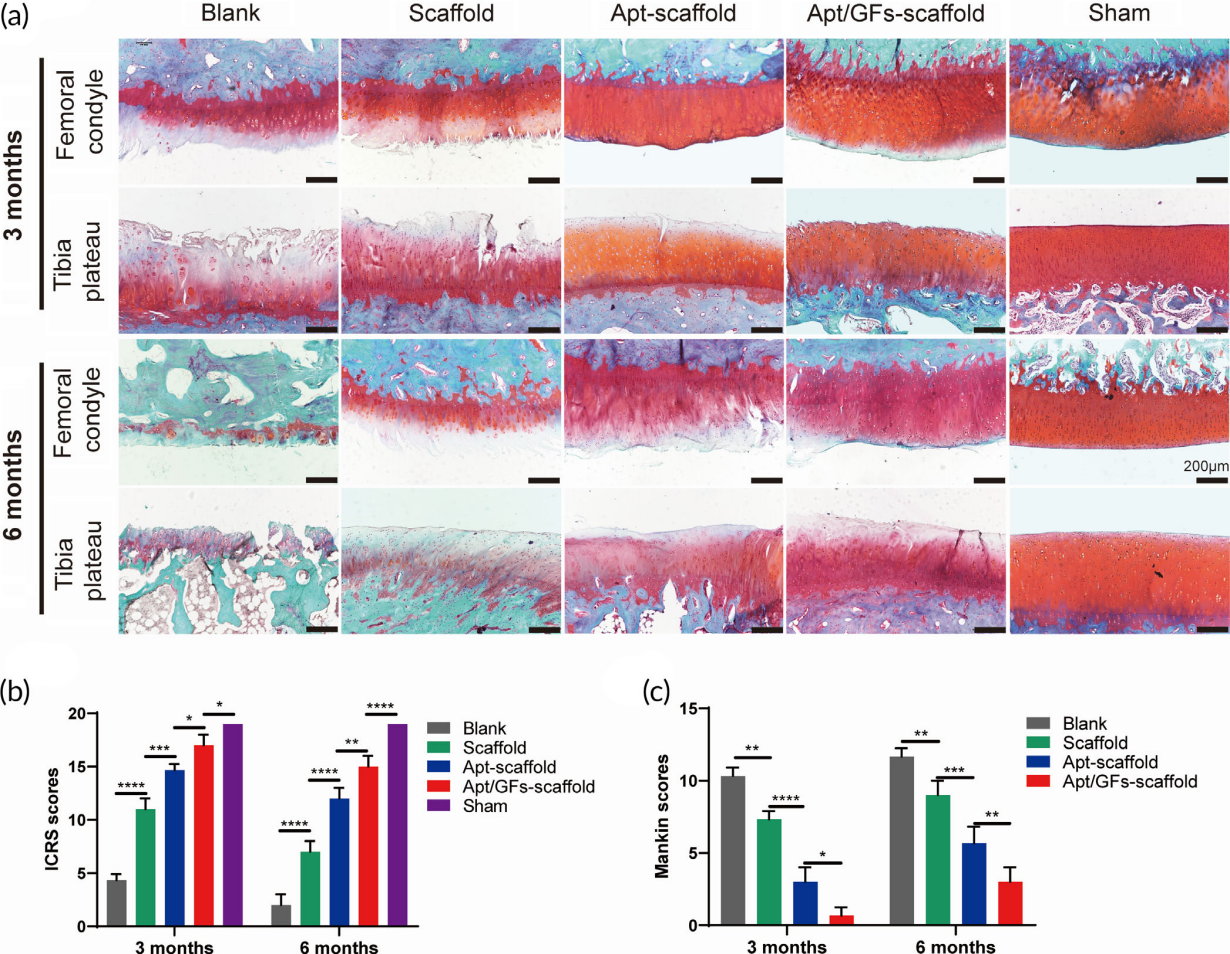
Safranin‐O/fast green (SOG) staining images (a), ICRS evaluation (b) and Mankin scores (c) for cartilage degeneration of the femoral condyle and tibial plateau (*n* = 3). Data are means ± SD. Two‐way analysis of variance in (b) and (c). ns, no significant difference, *p < 0.05, **p < 0.01, ***p < 0.005, ****p < 0.001

It was important to evaluate the chondroprotective effect of various scaffolds. Gross observation of cartilage (Figure 8a) showed less cartilage reduction and degradation in the Apt/GF‐scaffold group than in the other groups except for the sham group at each time point, which was also verified by histological analysis (Figure 10a). Indeed, the cartilage of the femoral condyle and tibial plateau exhibited complete disorganization and severe reductions in SOG staining in the blank group at 3 months and 6 months. The ICRS and Mankin scores also showed the worst cartilage performance over time in the blank group, followed by the scaffold group (Figure 10b,c). SOG staining showed obvious cartilage degradation and clefts in the blank and scaffold groups but seldom in the Apt‐scaffold group and the Apt/GF‐scaffold group, and the latter was better, demonstrating the superior cartilage protection ability of the Apt/GF‐scaffold group. Overall, the blank group showed deep cracks and lacunae in the femoral condyle and tibial plateau. The scaffold group and the Apt‐scaffold group also exhibited superficial cracks and tangled fibrous surfaces, while in the Apt/GF‐scaffold group, the cartilage had a flatter surface with scarcely any cracks.

## 4 DISCUSSION

The meniscus is an anisotropic tissue that possesses limited innate capacity to mount a sufficient healing response, especially in the inner region.^4^ Hence, regenerating the meniscus and further protecting articular cartilage from degeneration and the occurrence of symptomatic OA present a Gordian knot for clinicians and bioengineering scientists. Autologous regeneration may be useless if a meniscus is torn, and the traditional treatment for a meniscus defect is arthroscopic meniscectomy or MAT.^2^ However, arthroscopic meniscectomy inevitably causes progressive cartilage degeneration, and MAT suffers from insufficient donors and disease transmission risk and requires more evidence to prove its chondroprotective effects.^31^, ^32^, ^33^ In the search for new approaches for meniscal regeneration, in situ tissue engineering has become a powerful strategy, and recent advances in methods and materials have provided opportunities to develop suitable constructs to mobilize ESPCs to the site of injury and foster a meniscogenic environment, eventually facilitating clinical repair of the injured meniscus. In this study, we aimed to develop an integrated bioactive scaffold for initial ESPC homing through the delivery of the aptamer Apt19S followed by manipulation of the migrated cells to differentiate into a fibrochondrogenic lineage via sequentially released GFs. To implement the designed scheme, integrated PCL/PDA/GE scaffolds were fabricated to incorporate PLGA microspheres/nanospheres encapsulated with TGF‐β3 and CTGF, and the prepared scaffolds physically absorbed Apt19S to achieve a bioactive cell‐free scaffold (Apt/GF‐scaffold) for improved meniscal regeneration and superior chondroprotective effects.

A bioactive scaffold applied for meniscal tissue engineering should primarily provide interconnecting and complex microchannels for endogenous cell adhesion and infiltration, as well as favorable physical strength to maintain biomechanical supports. Recently, a promising strategy has emerged to construct biomimetic meniscal implants by utilizing synthetic polymers as a supporting backbone, while natural polymers serve as an additive element to mimic extracellular microenvironments.^2^ The decellularized meniscal tissue developed in our laboratory was used to fabricate implantable meniscal substitutes. In contrast to other natural polymers, MECM was made through physical–chemical methods to retain the bioactivity of the native tissue during fabrication, thus possessing the advantage of better‐matched meniscal biological properties.^25^ However, considering the fast degradation and unfavorable strength, MECM was blended with GelMA to match the requirements of meniscal tissue engineering scaffolds and played a role in creating an optimized structure and microenvironment for accelerating fibrochondrogenesis inductive matrix synthesis.^34^ Despite the biochemical properties of scaffold materials, previous studies have proven that the microarchitectures of tissue‐engineered scaffolds are also key factors for cell behaviors (e.g., cell proliferation, differentiation, and ECM synthesis).^35^ It has been demonstrated that in meniscal regeneration, optimal scaffold architecture (e.g., average pore size) is a prerequisite for ideal repair outcomes.^36^ In our study, we achieved optimized macro‐microporous structures of scaffolds with pore sizes between 60 and 220 μm (Figures S1 and S2), considering the similar components compared to collagen scaffolds, which were believed to meet the needs of cell ingrowth, proliferation, and differentiation in both dry and hydrated conditions.^23^, ^37^ However, further studies are needed to investigate whether the pore sizes in our scaffold are stable, as the SEM images could not truly reflect the real pore size of hydrated scaffolds, and the influence of this impairment on cell infiltration was caused by reduced connectivity in hydrated conditions requires further tests. At the other end of the spectrum, polymer PCL was 3D printed as a backbone to support the ECM‐like hierarchical structure, as the mechanical characteristics of this ECM‐based material are not able to meet the requirements of the native menisci.^23^ In addition, with the subsequent PDA modification, the PCL scaffolds with a PDA layer exhibited advantages in meniscal repair, such as rougher surface and hydrophilic properties (Figure 2b,d), and may possess prochondrogenic ability, reduced foreign body reaction, and immunomodulation effects.^13^, ^38^

Retaining migrated cells at the meniscus defect site and promoting their infiltration and spreading are additional key prerequisites of meniscal scaffolds. In our study, the results of the dynamic contact angle test indicate that PDA modification and GE introduction significantly increase the hydrophilicity compared with the pure PCL scaffold (Figure 2d), which can give a more favorable microenvironment for cell adhesion. Moreover, these components and modifications have been proven to have a positive impact on SMSC viability, as demonstrated by the live/dead (Figure 3a) and CCK‐8 assays (Figure 3d). The in vitro DAPI/F‐actin staining studies also showed that the hybrid scaffold with appropriate microarchitecture and adhesion cues enabled the SMSCs to adhere to typical fusiform morphology and distribute better and larger compared with the pure PCL scaffold (Figure 3b,c). Collectively, these physicochemical traits endow scaffolds with an interconnected porous structure, excellent surface, and bioactivity and further provide a biomimetic microenvironment for cell adhesion, migration, and proliferation.

It has been well established that sufficient ESPCs are crucial for the meniscal healing process; however, the inherent recruitment and mobilization of ESPCs to the injured sites are inadequate for the repair of meniscal injury.^29^, ^30^, ^39^ Although some chemokines and GFs, such as stromal cell‐derived factor‐1 and platelet‐derived GF‐BB, have been used to promote ESPC migration toward meniscal injury sites, the nonspecific mobilization induced by these factors, including the migration of proinflammatory or endothelial cells, might excessively activate inflammatory responses and thus impede meniscal regeneration.^40^, ^41^ Therefore, it is important to discover a chemoattractant that can specifically recognize and efficiently recruit ESPCs for endogenous meniscal regeneration. In recent years, some researchers reported that the aptamer Apt19S could specifically recognize and bind to MSCs and thus promote osteochondral and chondral repair when immobilized in scaffolds.^15^, ^42^ Wang et al. reported that aptamer‐functionalized NPs enhanced bone defect repair by improving stem cell recruitment.^43^ Similarly, our previous study found that the aptamer HM69‐encapsulated 3D bioprinting scaffold achieved specific stem cell recruitment in vivo and promoted in situ articular cartilage regeneration. Based on these findings, in our studies, we chose the aptamer Apt19S to innovatively design an aptamer‐targeted multifunctional scaffold that might be able to specifically and effectively recognize, bind, and recruit ESPCs to meniscal injury sites. First, the binding affinity and specificity of Apt19S on SMSCs were investigated, and the in vitro results in Figure 4a,b showed that FAM‐labeled Apt19S could specifically recognize and bind to rabbit SMSCs but not rabbit MFCs or mouse macrophages. Subsequently, we discovered that Apt19S at concentrations of 10 nM and 100 nM could significantly promote SMSC migration in vitro but showed no effects on other types, including MFCs and macrophages (Figure 4c–g, Figures S6 and 7). Apt19S at a concentration of 1 nM showed no promoting effects on SMSC migration; however, with increasing concentrations of Apt19S, the migration of SMSCs was observed. Notably, the number of migrated SMSCs in the 10 nM and 100 nM groups showed no significant difference, indicating that 10 nM Apt19S might be sufficient for enhancing MSC migration. After that, Apt19S was chemically conjugated to the MECM sponge, and confocal imaging revealed that FAM‐labeled Apt19S was well distributed in the MECM sponge (Figure S8). We then further determined ESPC recruitment by injecting the Apt19S‐functionalized hydrogel into rat meniscal defects, and the samples were harvested and stained by immunofluorescence 1 week postsurgery. The in vivo results in Figure 5 revealed that Apt19S in the hydrogel could significantly enhance ESPC migration toward meniscal injury sites, whereas a single hydrogel or GFs did not exhibit such effects. In summary, we systematically investigated the binding specificity and affinity and recruitment effects of Apt19S on SMSCs in vitro and in vivo, paving the way for in situ meniscal regeneration.

In addition to recruiting sufficient and specific ESPCs to meniscal injury sites, regional differentiation of the meniscus is another insurmountable barrier that remains to be addressed.^19^ The rationale for choosing CTGF for meniscal regeneration was related to its potent regulation of the gene expression of fibrochondrogenesis, including COL1, TNC, and FN1. Meanwhile, TGF‐β3 is a powerful factor in inducing chondrogenic differentiation of MSCs. As previously noted, the sequential delivery of CTGF and TGF‐β3 was proven to promote MSC differentiation into fibrochondrocytes in vitro and in vivo, which helped to rebuild heterogeneous meniscal tissue phenotypes of the meniscus.^19^ Hence, CTGF and TGF‐β3 were incorporated into PLGA NPs and MPs, respectively, and the in vitro GF release profiles showed that CTGF was released faster than TGF‐β3, which might meet the requirement for inducing heterogeneous MSC differentiation (Figure 2h). The in vitro results showed that the introduction of two GFs could significantly enhance chondrogenic and fibrochondrogenic differentiation after 7 days of culture of SMSCs. Additionally, as the culture time was extended to 14 days, the level of fibrochondrogenic genes, except for TNC expression, exhibited further improvement (Figure 6). Upon statistical analysis, as shown in Figure 6, a significant synergistic effect was achieved by the temporal release of CTGF and TGF‐β3 in vitro, indicating promise for inducing in vivo anisotropic differentiation for meniscal regeneration.

Rabbits have been frequently chosen as translational animal models due to their small size and lower cost.^44^ The MRI image results demonstrated more meniscus‐like tissue formation (Figure 7a) and lower WORMS scores (Figure 7b) in the Apt/GF‐scaffold group at both 3 months and 6 months post‐operation. In the blank group, there was no tissue regeneration, which may demonstrate the extreme scarcity of the endogenous repair capacity of the meniscus to regenerate without rigorous intervention. For the experimental groups, better meniscus‐like tissue was observed and covered the tibial plateau cartilage well (Figure 8a), while there were significant differences in the size, shape, histological structure, and biochemical contents of the regenerated menisci in these groups. Although the neomeniscus could be observed in the scaffold group, it was inferior to that in the Apt‐scaffold group and the Apt/GF‐scaffold group. We speculate that the hybrid scaffold could only provide structural support and a favorable microenvironment for tissue ingrowth but lacked sufficient biological activity to recruit ESPCs both in quality and quantity, which signified that this cell‐free scaffold could not strongly orchestrate the repair process of the defective meniscus. Hence, these findings indicated the necessity of bioactive factors in the meniscal regeneration process. In general, the regenerated menisci in the Apt/GF‐scaffold group were superior to those in the Apt‐scaffold group in terms of histological structure and biochemical contents at 3 and 6 months after implantation, which emphasized the potency of the combination of bioactive factors for meniscal regeneration. Numerous migrated ESPCs induced by Apt19S may only quickly differentiate to form fibrous tissue filled with defects in the posttraumatic intrajoint environment but potent stimuli to initiate the tissue remodeling process, which is of utmost importance for meniscal regeneration, are lacking. Therefore, the introduction of sequentially released GFs (e.g., CTGF and TGF‐β3) may represent an efficient strategy to improve the quality of regenerated tissue. The macroscopic (Figure 8a) view and histological staining collectively demonstrated better meniscal regeneration in the Apt/GF‐scaffold group than in the sham group. Significantly more cell infiltration and production of GAG, outer regional COL I, and inner regional COL II were also observed in the Apt/GF‐scaffold group by H&E, TB, SR, immunohistochemistry staining (Figure 9a–f), and biochemical analysis (Figure 8b–g), which demonstrated that this combinatorial strategy including targeted cell recruitment and directional differentiation possessed a strong ability to orchestrate the staged regeneration process to achieve improved meniscal regeneration with heterogeneous deposition of collagens and proteoglycans.

In terms of the chondroprotective effect, different levels of cartilage damage were detected. The regenerated meniscus in the Apt/GF‐scaffold group was shown to be more effective in preventing cartilage degeneration than those in the Apt‐scaffold and scaffold groups, which was consistent with the quality of the regenerated meniscus (Figure 10), ICRS scores (Figure 10b) and Mankin scores (Figure 10c). Because the meniscus exerts a protective effect on the cartilage surface by allowing load transmission, stabilization, and shock absorption,^45^ the inferior size and biomechanical properties of the regenerated menisci lead to a weakened chondroprotective effect.^46^ A previous study also reported that removal of 30%–60% of the medial meniscus increased compressive strain to approximately 10%–20%, which could accelerate cartilage degeneration and OA progression.^47^ Therefore, cartilage damage was evident in the positive control group due to instability of the knee joint and the increased loading imposed on articular cartilage without the meniscus.

Despite these results, the structural and functional properties of the PCL‐based constructs were still slightly inferior to those of the native meniscus. Some parameters, such as the scaffold material and factors, may require further evaluation to improve the structure (e.g., pore size may influence cell infiltration) and mechanical properties of the regenerated meniscus. In addition, we expect that the degradation of the PCL scaffold can be extended to 9–12 months. In this way, an adequate ECM can maintain biomechanical support during tissue ingrowth. Finally, the PCL‐based scaffold could degrade and become remodeled in the tissue‐engineered meniscus. The major limitation of the present study is that we could not follow the fate of endogenous or exogenous stem/progenitor cells within the knee joint in vivo, which would serve to interpret the mechanisms underlying cell proliferation and differentiation. Although tracking cell migration and differentiation is challenging, future studies may utilize green fluorescent protein or other genetically encoded labeling methods to achieve this goal.^48^ We also defined the inner and outer regions of the meniscus as a measure of anisotropy, but more stringent models may be needed to consider the gradual spatial transition of the heterogeneous phenotypes and ECM compositions of the native meniscus. Furthermore, large animal models (sheep, goat, or primate) may be more relevant to future studies concerning the development of clinical treatments.

## 5 CONCLUSION

An in situ staged meniscal regeneration strategy that combines targeted ESPC recruitment and directed profibrochondrogenesis is a promising approach for the regeneration of injured meniscal tissue. As a proof of concept, we presented an interconnected porous 3D PCL/PDA/GE‐Apt/GF‐scaffold with biomimetic microarchitecture and incorporated three factors to sequentially activate ESPC homing and fibrochondrogenic differentiation in a two‐stage manner, subsequently achieving superior meniscal regeneration and articular cartilage protection in a rabbit major medial meniscectomy model. The primary stage involved the fast release of Apt19S, which acted as a signaling molecule to stimulate the mobilization and recruitment of resident ESPCs within the joint to infiltrate into the scaffold. In the second stage, sequentially released CTGF and TGF‐β3 cooperated with biomechanical stimuli to direct the fibrochondrogenic differentiation of migrated and proliferated cells in the targeted space. Moreover, the ECM‐based scaffold proved to be an advantageous platform to guide ESPC adhesion, migration, and proliferation. These results not only provide a potential substitute that can guide meniscal tissue regeneration but also a promising ready‐made cell‐instructive meniscal product for clinical use.

## AUTHOR CONTRIBUTIONS

**Hao Li:** Conceptualization (equal); data curation (equal); formal analysis (equal); investigation (equal); writing – original draft (equal). **Tianyuan Zhao:** Conceptualization (equal); data curation (equal); formal analysis (equal). **Fuyang Cao:** Investigation (equal); methodology (equal); visualization (equal); writing – original draft (equal). **Haoyuan Deng:** Methodology (equal); resources (equal); software (equal); validation (equal). **Songlin He:** Formal analysis (equal); investigation (equal); validation (equal); visualization (equal). **Jianwei Li:** Methodology (equal); project administration (equal); software (equal); writing – original draft (equal). **Shuyun Liu:** Funding acquisition (equal); writing – original draft (equal); writing – review and editing (equal). **Zhiguo Yuan:** Conceptualization (supporting); resources (lead). **Quanyi Guo:** Conceptualization (lead); funding acquisition (lead); methodology (supporting); supervision (lead); writing – original draft (supporting).

## CONFLICTS OF INTEREST

All authors involved in this article declare that there are no conflicts of interest regarding the publication of this paper.

6

### PEER REVIEW

The peer review history for this article is available at https://publons.com/publon/10.1002/btm2.10302.

## Supporting information


**Figure S1** Pore size distribution of PCL/PDA/GE scaffold.

**Figure S2** Pore size distribution of PCL/PDA/GE‐Blank PLGA scaffold.

**Figure S3** SEM image of PLGA nanoparticles (NPs).

**Figure S4** SEM image of PLGA microparticles (MPs).

**Figure S5** Three peaks at 289.0, 286.5 and 285.0 eV in the C 1s spectrum can be observed, which attributed to O‐C=O, C‐O and C‐C groups of PCL, respectively.^9^ With the introduction of PDA, two new peaks of C 1s appeared, one was N‐C=O (288.57 eV), and the other was C‐N (286.8 eV), which was overlapped with C‐C. By further modification with GE and PLGA, there were no obvious new peaks in the C 1 s spectrum, while the intensity of each peak of the C1s mentioned above has changed.

**Figure S6** Statistical analysis and crystal staining (A‐D) of MFCs migration toward the (A) control group, (B) 1 nM **Apt19S**, (C) 10 nM **Apt19S**, and (D) 100 nM **Apt19S** in a Transwell system (n=5). Data are means ± SD. ns, means no significant difference.

**Figure S7** Statistical analysis and crystal staining (A‐D) of macrophages migration toward the (A) control group, (B) 1 nM **Apt19S**, (C) 10 nM **Apt19S**, and (D) 100 nM **Apt19S** in a Transwell system (n=5). Data are means ± SD. ns, means no significant difference

**Figure S8** Confocal images of FAM‐labeled **Apt19S** distribution in MECM sponge.

**Appendix S1**: Supporting Information.

## Data Availability

The datasets used and/or analyzed during the current study are available from the corresponding author on reasonable request.
