# Supplementary figures and images for: Integrated bioactive scaffold with aptamer‐targeted stem cell recruitment and growth factor‐induced pro‐differentiation effects for anisotropic meniscal regeneration
Source: Bioeng Transl Med. 2022 Mar 3;7(3):e10302. doi: 10.1002/btm2.10302 (PMC9472018; doi:10.1002/btm2.10302)

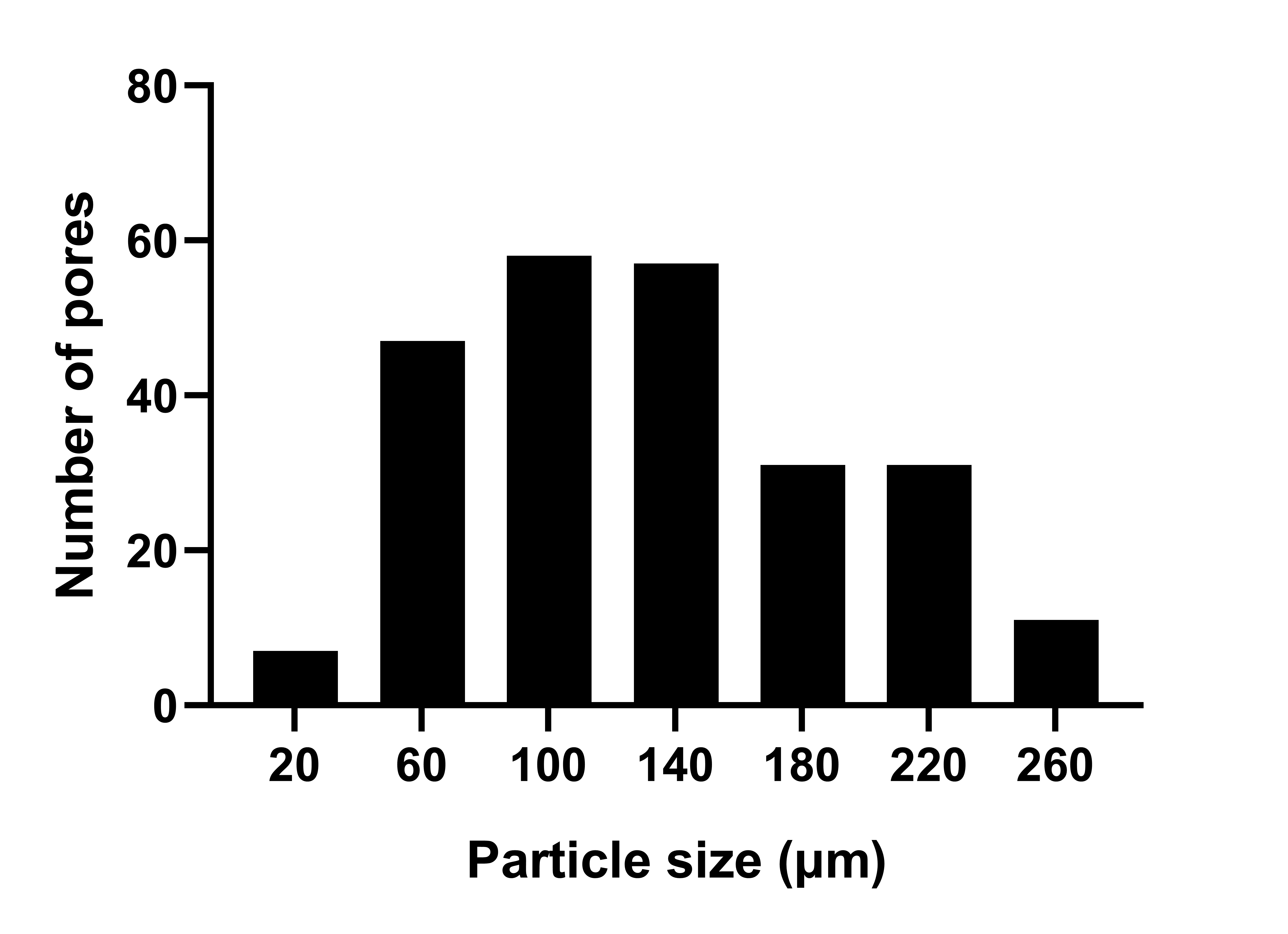

Supplement: Supplementary file 1 — Figure S1 Pore size distribution of PCL/PDA/GE scaffold. [file BTM2-7-e10302-s006.tif]

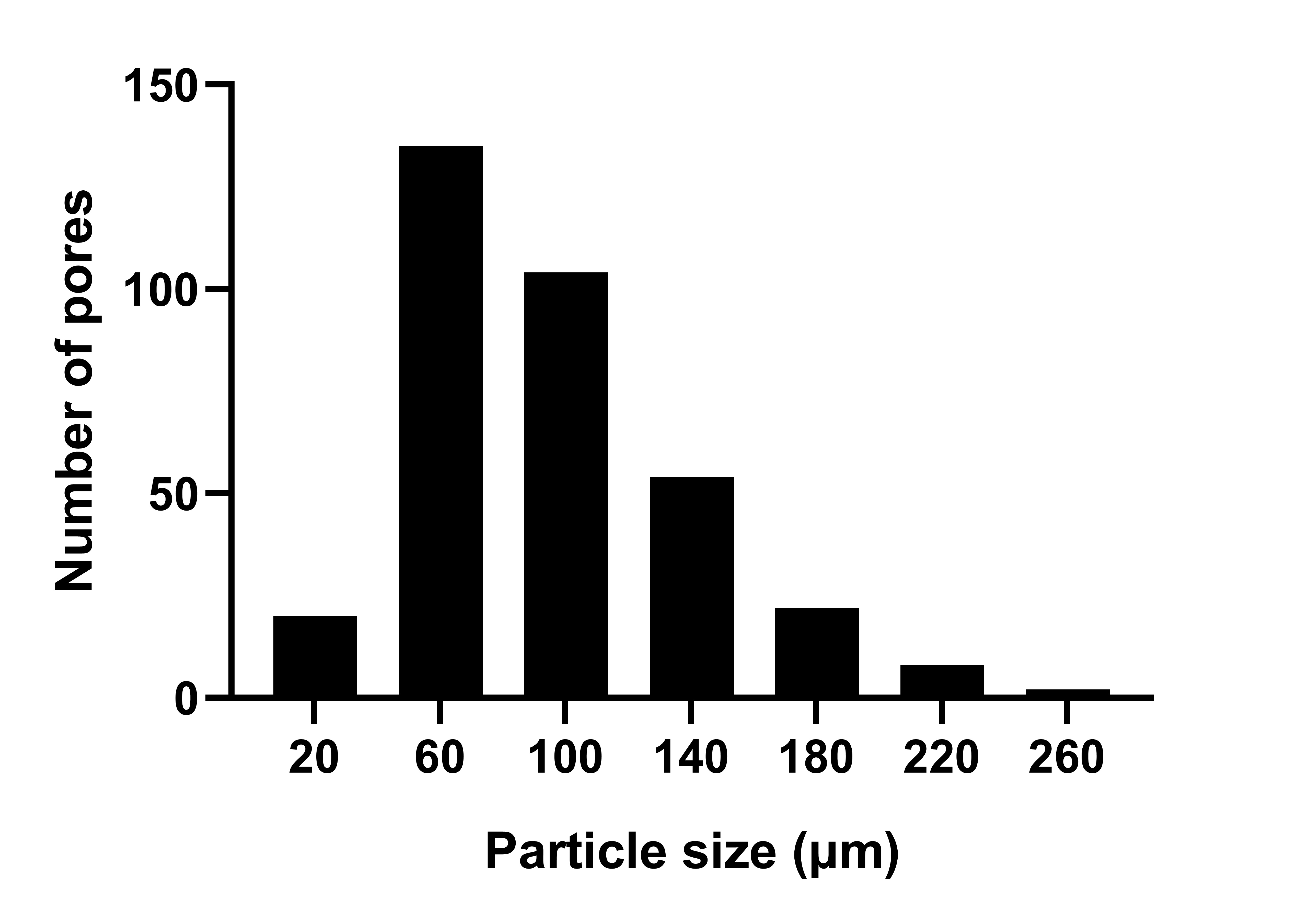

Supplement: Supplementary file 2 — Figure S2 Pore size distribution of PCL/PDA/GE‐Blank PLGA scaffold. [file BTM2-7-e10302-s002.tif]

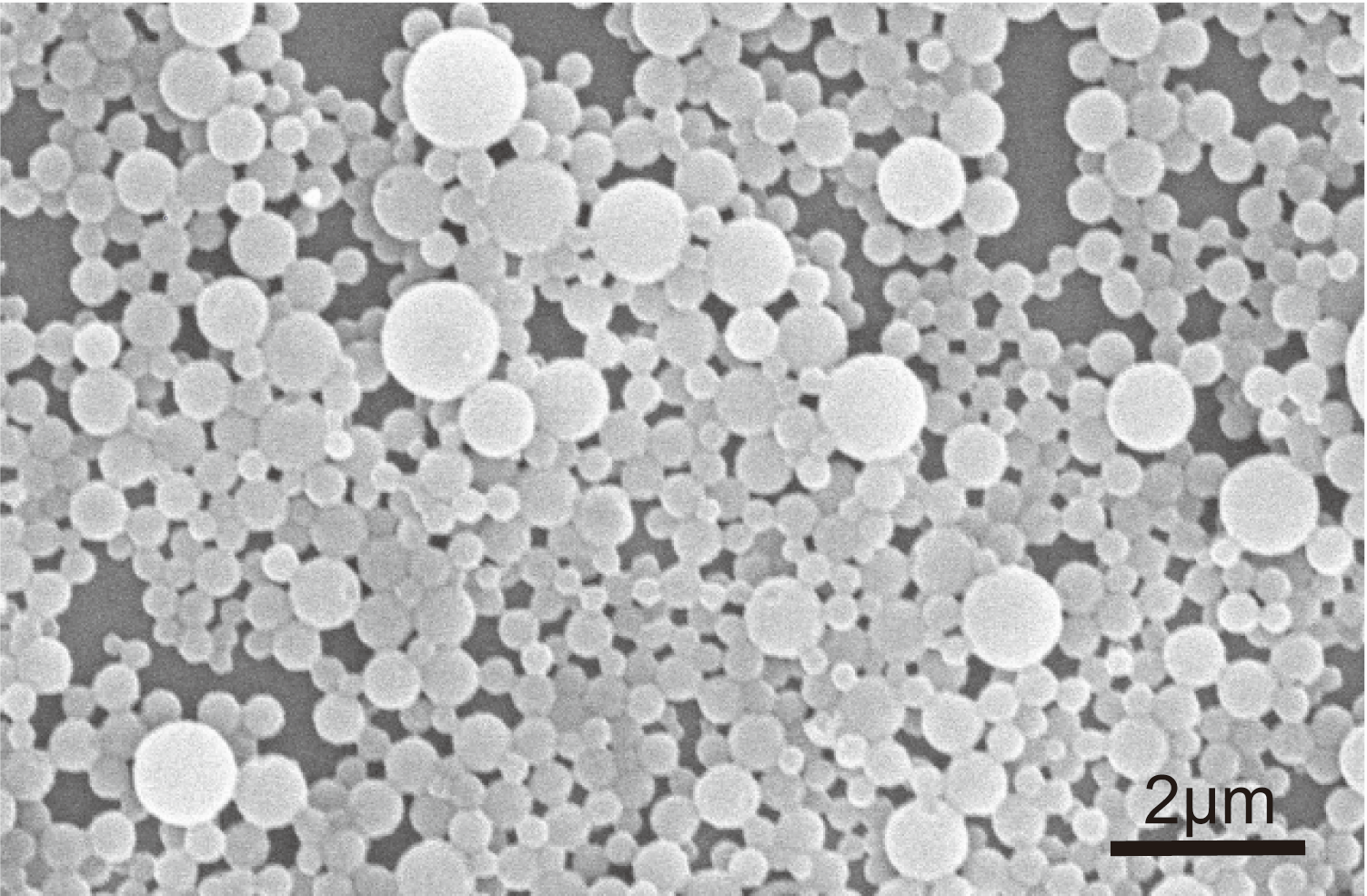

Supplement: Supplementary file 3 — Figure S3 SEM image of PLGA nanoparticles (NPs). [file BTM2-7-e10302-s007.png]

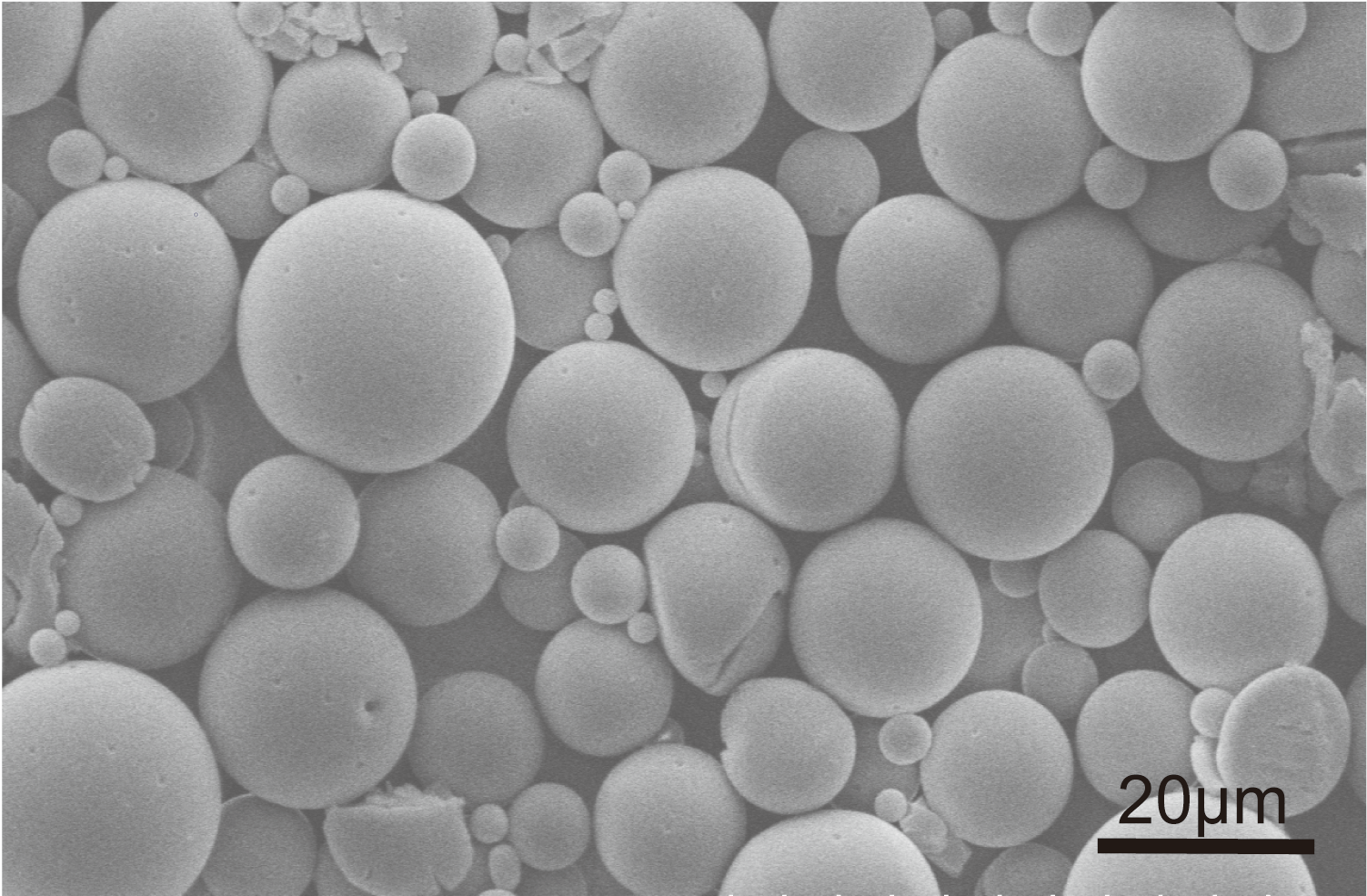

Supplement: Supplementary file 4 — Figure S4 SEM image of PLGA microparticles (MPs). [file BTM2-7-e10302-s009.png]

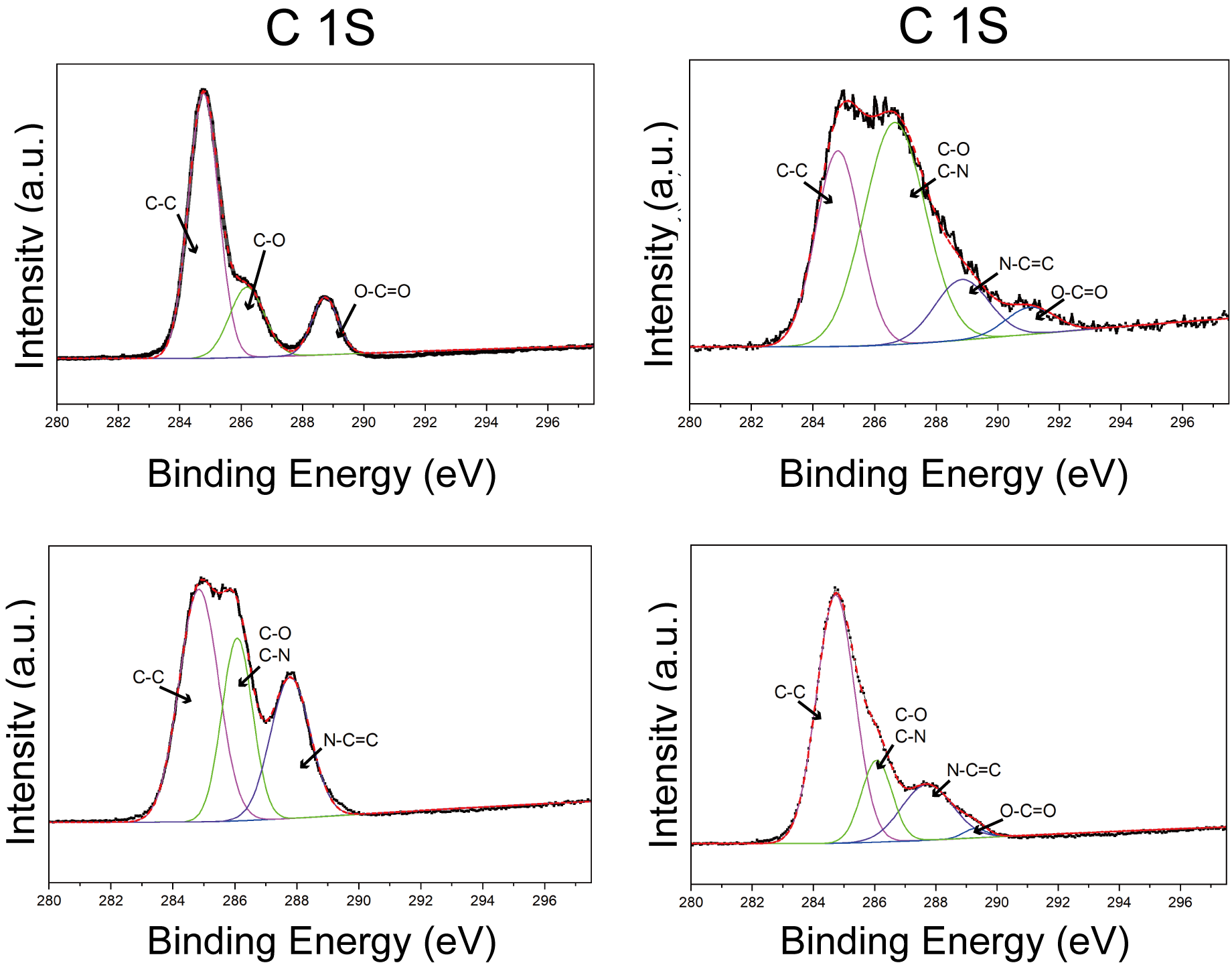

Supplement: Supplementary file 5 — Figure S5 Three peaks at 289.0, 286.5 and 285.0 eV in the C 1s spectrum can be observed, which attributed to O‐C=O, C‐O and C‐C groups of PCL, respectively.9 With the introduction of PDA, two new peaks of C 1s appeared, one was N‐C=O (288.57 eV), and the other was C‐N (286.8 eV), which was overlapped with C‐C. By further modification with GE and PLGA, there were no obvious new peaks in the C 1 s spectrum, while the intensity of each peak of the C1s mentioned above has changed. [file BTM2-7-e10302-s003.png]

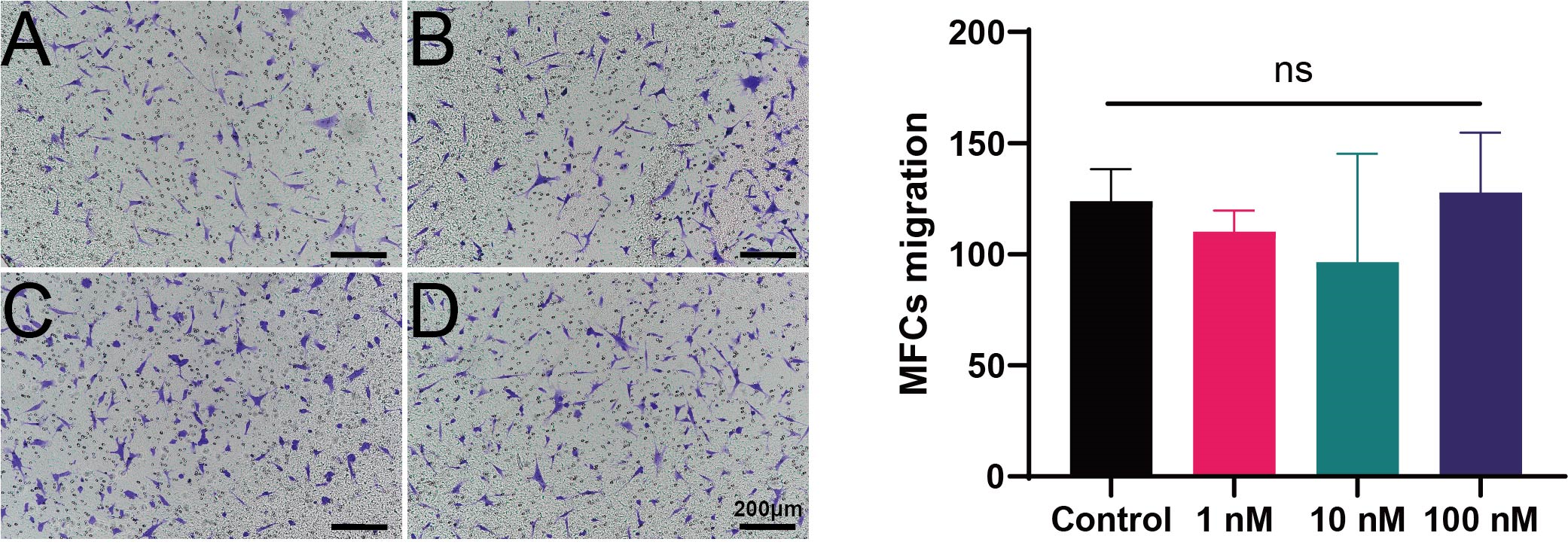

Supplement: Supplementary file 6 — Figure S6 Statistical analysis and crystal staining (A‐D) of MFCs migration toward the (A) control group, (B) 1 nM Apt19S, (C) 10 nM Apt19S, and (D) 100 nM Apt19S in a Transwell system (n=5). Data are means ± SD. ns, means no significant difference. [file BTM2-7-e10302-s005.png]

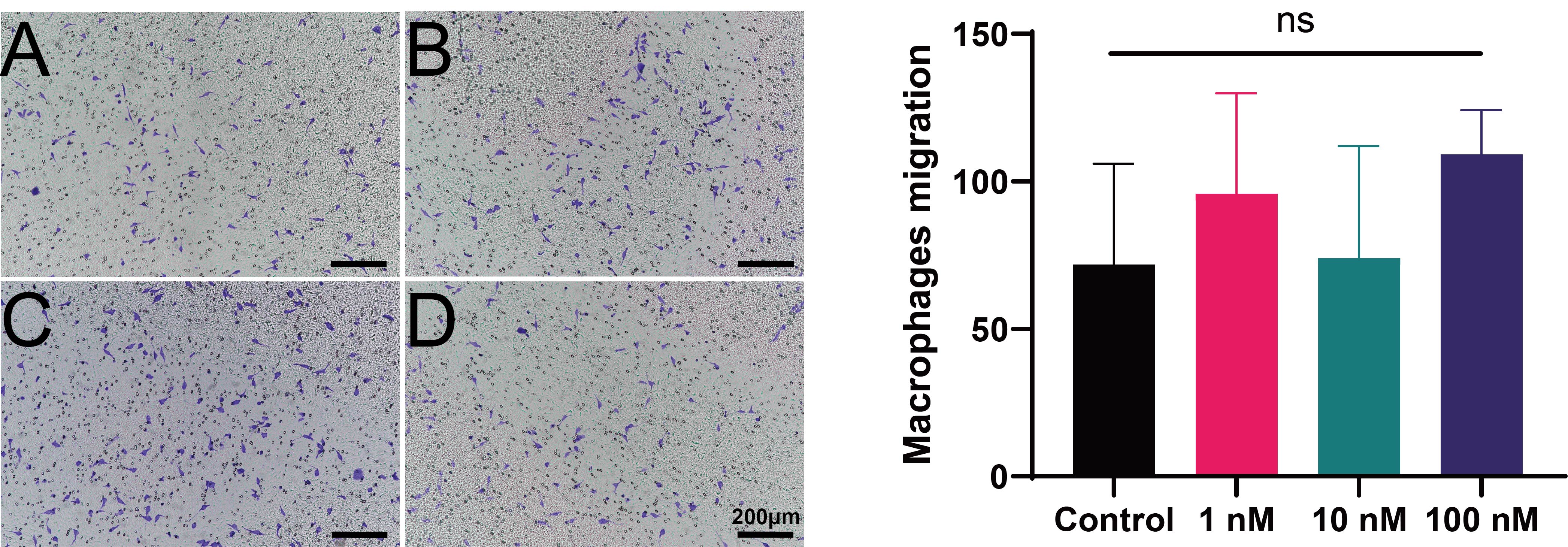

Supplement: Supplementary file 7 — Figure S7 Statistical analysis and crystal staining (A‐D) of macrophages migration toward the (A) control group, (B) 1 nM Apt19S, (C) 10 nM Apt19S, and (D) 100 nM Apt19S in a Transwell system (n=5). Data are means ± SD. ns, means no significant difference [file BTM2-7-e10302-s001.jpg]

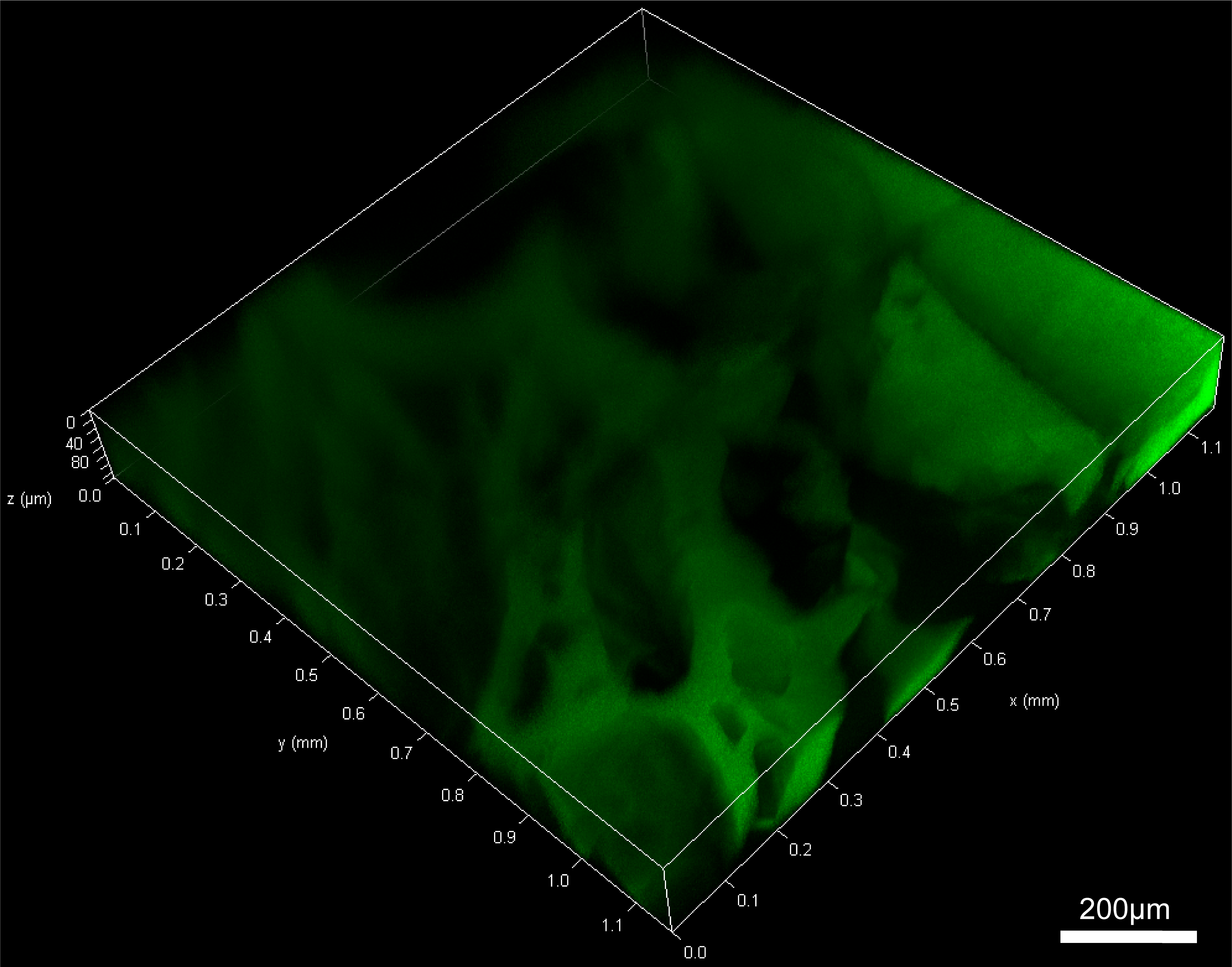

Supplement: Supplementary file 8 — Figure S8 Confocal images of FAM‐labeled Apt19S distribution in MECM sponge. [file BTM2-7-e10302-s004.png]
