# Supplementary material for: Integrated bioactive scaffold with aptamer‐targeted stem cell recruitment and growth factor‐induced pro‐differentiation effects for anisotropic meniscal regeneration
Source: Bioeng Transl Med. 2022 Mar 3;7(3):e10302. doi: 10.1002/btm2.10302 (PMC9472018; doi:10.1002/btm2.10302)
Supplement: Supplementary file 9 — Appendix S1: Supporting Information. [file BTM2-7-e10302-s008.docx]

**Supplementary material**

**Integrated bioactive scaffold with Aptamer-targeted stem cell recruitment and growth factors-induced pro-differentiation for anisotropic meniscal regeneration**

Li Hao^1,2#^, Zhao Tianyuan^1,2^^#^, Cao Fuyang^1,3#^, Deng Haoyuan^1,2^, He Songlin^1,2^, Li Jianwei^1,2^, Ma Yang^1^, Wang Yue^1^, Liu Shuyun^1,2^ , Yang Zhen^1,2,4^*, Yuan Zhiguo^1,5^*, Guo Quanyi^1,2^*

1: Institute of Orthopedics, the First Medical Center, Chinese PLA General Hospital; Beijing Key Lab of Regenerative Medicine in Orthopedics; Key Laboratory of Musculoskeletal Trauma & War Injuries PLA; No. 28 Fuxing Road, Haidian District, Beijing 100853, China

2: School of Medicine, Nankai University, Tianjin 300071, China

3: Department of Orthopedics, the First Affiliated Hospital of Zhengzhou University, 1 Jian East Road, Eqi District, Zhengzhou 450052, China

4: Arthritis Clinic & Research Center, Peking University People's Hospital, Peking University, Beijing 100044, China

5: Department of Bone and Joint Surgery, Renji Hospital, School of Medicine, Shanghai Jiaotong University, Shanghai, China

***: Corresponding authors:**

(1) Guo Quanyi, Institute of Orthopedics, The First Medical Center, Chinese PLA General Hospital, Beijing Key Lab of Regenerative Medicine in Orthopedics, Key Laboratory of Musculoskeletal Trauma and War Injuries PLA, No. 28 Fuxing Road, Haidian District, Beijing 100853, China. School of Medicine, Nankai University, Tianjin 300071, China. Email address: doctorguo_301@163.com.

(2) Yuan Zhiguo, Department of Meniscus and Joint Surgery, Renji Hospital, School of Medicine, Shanghai Jiaotong University, Shanghai, China. Email address: yzgad@163.com.

(3) Yang Zhen, Arthritis Clinic & Research Center, Peking University People's Hospital, Peking University, Beijing 100044, China. Email address: zhenyang_pku@163.com

#: These authors contributed equally.

**1. Materials and methods**

1.1. Preparation of the printable MECM

Decellularized MECM slurry was prepared by physical-chemical method from the porcine meniscal tissue as described previously [1-3]. Briefly, the lateral and medial menisci were harvested from porcine cadaveric joint and sterilizing with 3% hydrogen peroxide (H_2_O_2_, Sigma-Aldrich, United States). The pulverizing menisci were treated with pepsin and acetic acid (Sigma Aldrich, United States), then homogenized at 4 ℃. Meniscus tissue was decellularized according to the differential centrifugation methods adopted by 2000 rpm for 10 min, 6000 rpm for 30 min, and 10000 rpm for 30 min. These procedures were repeated 5 times to achieve decellularization, and the obtained MECM slurry was stored at 4℃.

1.2. Fabrication of the CTGF loaded PLGA nanoparticles (NPs) and TGF-β3 loaded PLGA microparticles (MPs)

CTGF NPs were prepared through double-emulsion solvent evaporation method according to a previous report [4]. Briefly, 60 mg of PLGA (75:25) was dissolved into 1ml of dichloromethane followed by adding 1ml mixed solution (dimethyl sulfoxide (DMSO): dichloromethane=1:4) containing 4.8 μg of CTGF. Then, this primary emulsion was emulsified by ultrasonicating for 3 minutes (power of 75W) to reduce the size of PLGA particles. After this, the primary solution was added into 20 ml 1% (w/v) poly vinyl alcohol (PVA) solution to form second emulsion by magnetically stirred for 12 h to evaporate the solvent. Then, the emulsion was filtrated, washed with distilled water for 5 times, and then lyophilized to obtain the nanoparticles, and stored at 4 ℃. For TGF-β3 MPs, PLGA MPs encapsulating TGF-β3 were prepared by a modified double-emulsion technique [3, 5].TGF-β3 (1.2 μg) was diluted to 1 ml mixed solution following the protocols above and added to the PLGA solution, forming primary emulsion that was emulsified via an ultrasonic homogenizer for 30 s at an same power. The primary emulsion was then added to 20 ml of 1% PVA, followed by same evaporation, wash and lyophilization procedures. And the GFs-free PLGA particles were fabricated following the same protocols.

1.3 Isolation and culture of rabbit SMSCs and rabbit meniscal fibrochondrocytes (MFCs)

SMSCs and MFCs were isolated from New Zealand white rabbits as described in previous studies [6, 7]. RAW 264.7 cells (murine macrophage line) were obtained from the American Type Culture Collection (Bethesda, MD, USA). The study on animals were approved by the Institutional Animal Care and Use Committee at PLA General Hospital. Briefly, cells were cultured in 25 cm^2^ flasks (Corning, USA) by adding Dulbecco's modified Eagle's medium/F12 (DMEM/F12, Corning, USA) containing 10% fetal bovine serum (FBS, Gibco, USA) and 1% penicillin-streptomycin (Sigma, USA). SMSCs and MFCs were passaged after cell fusion, and the 2nd-5th passages of cells were chosen to perform the following studies.

1.4 Cell viability and morphology

Each scaffold including PCL, PCL/PDA, PCL/PDA/GE and PCL/PDA/GE-Blank PLGA scaffold was seeded with 5×10^5^ SMSCs in 20 μl of DMEM/F12 (10% FBS) media after it was sterilized with cobalt-60 and allowed to adhere for 90 min, during which 20 μl of medium was changed every 30 min; subsequently, fresher medium was added and changed every 2 d over the next 4 d. To confirm the viability of SMSCs seeded on the scaffold, Live/Dead staining was performed after the scaffold-cell composites were harvested after 4 d of culturing. Briefly, the scaffold-cell composites were gently washed with fresh sterile PBS buffer and then immersed in 2 ml of PBS working solution with 4 mM ethidium homodimer-1 and 2 mM calcein-AM for 30 min at room temperature. Excitation wavelengths of 556 nm and 488 nm were used with a Leica TCS-SP8 confocal microscope (Leica, Germany) to detect the visualization of ethidium homodimer-1 (red color represents dead cells) and calceinAM (green color represents live cells). In addition, the morphology of SMSCs on scaffolds was assessed by staining with 4',6-diamidino-2-phenylindole (DAPI) and phalloidin (Cytoskeleton, USA) after 4 d of culture according to the manufacturer’s instructions. After the scaffold-cell composites were washed 3 times with fresh sterile PBS, the images were observed using a Leica TCS-SP8 confocal microscope.

1.5 ESPCs Recruitment Assay in vivo

Animal experiments were approved by the Institutional Animal Care and Use Committee at PLA General Hospital. The method of establishing the chondral defect model was described in our previous study [3, 8]. Briefly, animals were first anesthetized (10% chloral hydrate), shaved, and disinfected. The medial parapatellar approach was applied to expose the knee joints. The patella was laterally dislocated, and the knee was placed in full flexion. A cylindrical full-meniscus defect (1.5 mm diameter and 1 mm depth) was created with a dental drill on both limbs at the center of the meniscus. All debris was removed from the defect with curettage and irrigation.

1.6 Surgical procedures

The study on animals were approved by the Institutional Animal Care and Use Committee at PLA General Hospital. Thirty rabbits (male, 6 months old, 2.5-3.0 kg, n=6 knees for each group at single time point) were randomized into five groups (two knees of each rabbit were used): (a) the Native group (denoted as Sham group), (b) the Scaffold group, (c) the Apt-scaffold group, (d) the Apt/GFs-scaffold group and (e) the Blank group. The method of establishing the critical-size medial meniscectomy was described in our previous study [7]. Briefly, the animals were anesthetized, shaved, and disinfected. Then, an anteromedial parapatellar incision was created and the patella was luxated medially. After that, flexing the joint to exposure the medial meniscus and critical-size medial meniscectomy was made (only 5% of the external rim was left) and grafted with different scaffolds. The rabbits that underwent a sham operation were served as the Native group, those who underwent the only meniscectomy were served as the Blank group. For the experimental groups, the body of implant were sutured to the surround meniscal and synovium tissue, respectively. Finally, the knee joint of the rabbit was sutured in layers and the antibiotics were given intramuscularly to avoid infections. After surgery, there are not joint immobilization method for rabbits. After 3 and 6 months, the rabbits were sacrificed and the meniscus, femurs, and tibial plateaus were sampled for subsequent evaluation.

**2. Tables and figures**

**Table S1. The sequences of Apt19S and modified Apt19S used in this study.**

| Name | Sequence (5’-3) |
| --- | --- |
| **Apt19S** | AGGTCAGATGAGGAGGGGGACTTAGGACTGGGTTTATGACCTATGCGTG |
| Amino-labeled **Apt19S** | **NH_2_-[C]6-** AGGTCAGATGAGGAGGGGGACTTAGGACTGGGTTTATGACCTATGCGTG |
| FAM/amino-dual labeled **Apt19S** | **NH_2_-(C)_6_-** AGGTCAGATGAGGAGGGGGACTTAGGACTGGGTTTATGACCTATGCGTG **-FAM** |

Abbreviations: 5-carboxyfluorescein: FAM.

**Table S2 Primers designed for RT-qPCR.**

| Target genes |  | Sequences |
| --- | --- | --- |
| COL2 | F: 5′−3′  R: 3′−5′ | CACGCTCAAGTCCCTCAACA  TCTATCCAGTAGTCACCGCTCT |
| ACAN | F: 5′−3′  R: 3′−5′ | GGAGGAGCAGGAGTTTGTCAA  TGTCCATCCGACCAGCGAAA |
| SOX9 | F: 5′−3′  R: 3′−5′ | GCGGAGGAAGTCGGTGAAGAAT  AAGATGGCGTTGGGCGAGAT |
| COL1 | F: 5′−3′  R: 3′−5′ | GCCACCTGCCAGTCTTTACA  CCATCATCACCATCTCTGCCT |
| TNC | F: 5′−3′ | TCTCTGCACATAGTGAAAAACAATACC |
|  | R: 3′−5′ | TCAAGGCAGTGGTGTCTGTGA |
| FN1 | F: 5′−3′ | CAAGCCTGGTTGTTACGACA |
|  | R: 3′−5′ | AGGTTCAGGTTTACTCTCGC |
| GAPDH | F: 5′−3′  R: 3′−5′ | CAAGAAGGTGGTGAAGCAGG  CACTGTTGAAGTCGCAG |

**Table S3 WORMS scoring system for the fat-suppressed T2-weighted MRI images.**

| ITEMS | Scores |
| --- | --- |
| **Cartilage signal and morphology** |  |
| Normal thickness and signal | 0 |
| Normal thickness but increased signal on T2-weighted images | 1 |
| Partial-thickness focal defect <1 cm in greatest width | 2 |
| Full-thickness focal defect <1 cm in greatest width | 2.5 |
| Multiple areas of partial-thickness (Grade 2.0) defects intermixed with areas of normal thickness, or a Grade 2.0 defect wider than 1 cm but <75% of the region | 3 |
| Diffuse (≥75% of the region) partial-thickness loss | 4 |
| Multiple areas of full-thickness loss (grade 2.5) or a grade 2.5 lesion wider than1cm but <75% of the region | 5 |
| Diffuse (≥75% of the region) full-thickness loss | 6 |
| **Subarticular bone marrow abnormality** |  |
| None | 0 |
| <25% of the region | 1 |
| 25% to 50% of the region | 2 |
| >50% of the region | 3 |
| **Subarticular cysts** |  |
| None | 0 |
| <25% of the region | 1 |
| 25% to 50% of the region | 2 |
| >50% of the region | 3 |
| **Flattening or depression of the articular surfaces** |  |
| Normal | 0 |
| Mild | 1 |
| Moderate | 2 |
| Severe | 3 |
| **Osteophytes** |  |
| None | 0 |
| Equivocal | 1 |
| Small | 2 |
| Small-moderate | 3 |
| Moderate | 4 |
| Moderate-large | 5 |
| Large | 6 |
| Very large | 7 |
| **Synovial thickening and joint effusion** |  |
| Normal | 0 |
| <33% of maximum potential distention; | 1 |
| 33%–66% of maximum potential distention; | 2 |
| >66% of maximum potential distention. | 3 |
| **Loose bodies in the synovial cavity** |  |
| None | 0 |
| 1 loose body | 1 |
| 2 loose body | 2 |
| 3 or more loose bodies | 3 |
| **The medial and lateral meniscus** |  |
| Intact | 0 |
| Minor radial tear or parrot-beak tear; | 1 |
| Nondisplaced tear or prior surgical repair; | 2 |
| Displaced tear or partial resection; | 3 |
| Complete maceration/destruction or complete resection | 4 |
| **Ligament independent scores for ACL, PCL, MCL and LCL** |  |
| Intact | 0 |
| Torn | 1 |
| **Synovial cysts or bursal collections about the knee** |  |
| Small | 1 |
| Moderate | 2 |
| Large | 3 |

**Table S4 Histological ICRS scoring system for cartilage degeneration.**

| CARTILAGE EVALUATION | POINTS |
| --- | --- |
| **Thickness of neo-formed cartilage** |  |
| Similar to the surrounding cartilage | 3 |
| Greater than the surrounding cartilage | 2 |
| Less than the surrounding cartilage | 1 |
| No cartilage | 0 |
| **Joint surface regularity** |  |
| Smooth, intact surface | 3 |
| Surface fissures (<25% neo-surface thickness) | 2 |
| Deep fissures ((≥25% neo-surface thickness) | 1 |
| Complete disruption of the neo-surface | 0 |
| **Morphology of neo-formed surface tissue** |  |
| Exclusively articular cartilage | 4 |
| Mainly hyaline cartilage | 3 |
| Fibrocartilage (spherical morphology observed with≥75% of cells) | 2 |
| Only fibrous tissue (spherical morphology observed with<75% of cells) | 1 |
| No tissue | 0 |
| **Chondrocyte clustering** |  |
| 25-100%chondrocytes | 3 |
| <25% chondrocytes | 2 |
| No chondrocytes present (no cartilage) | 1 |
| None at all | 0 |
| **Chondrocyte and GAG content of neo-cartilage** |  |
| Normal cellularity with normal Safranin O staining | 3 |
| Normal cellularity with moderate Safranin O staining | 2 |
| Clearly less cells with poor Safranin O staining | 1 |
| Few cells with no or little Safranin O staining or no cartilage | 0 |
| **Chondrocyte and GAG content of adjacent cartilage** |  |
| Normal cellularity with normal Safranin O staining | 3 |
| Normal cellularity with moderate Safranin O staining | 2 |
| Clearly less cells with poor Safranin O staining | 1 |
| Few cells with no or little Safranin O staining or no cartilage | 0 |

**Table S5 Mankin scoring system for cartilage degeneration.**

| CARTILAGE EVALUATION | GRADE |
| --- | --- |
| **Structure** |  |
| Normal | 0 |
| Surface irregularities | 1 |
| Pannus and surface irregularities | 2 |
| Clefts to transitional zone | 3 |
| Clefts to radial zone | 4 |
| Clefts to calcified zone | 5 |
| Complete disorganization | 6 |
| **Cells** |  |
| Normal | 0 |
| Diffuse hypercellularity | 1 |
| Cloning | 2 |
| Hypocellularity | 3 |
| **Safranin O staining** |  |
| Normal | 0 |
| Slight reduction | 1 |
| Moderate reduction | 2 |
| Severe reduction | 3 |
| No dye noted | 4 |
| **Tidemark integrity** |  |
| Intact | 0 |
| Crossed by blood vessels | 1 |

**Table S6 The surface element percentage of scaffolds.**

| Group | Atomic concentration (%) | | |
| --- | --- | --- | --- |
|  | C | O | N |
| PCL | 74.34 | 23.7 | 1.95 |
| PCL/PDA | 70.92 | 23.15 | 5.94 |
| PCL/PDA/GE | 61.61 | 20.55 | 17.84 |
| PCL/PDA/GE-Blank PLGA | 69.36 | 20.39 | 10.26 |


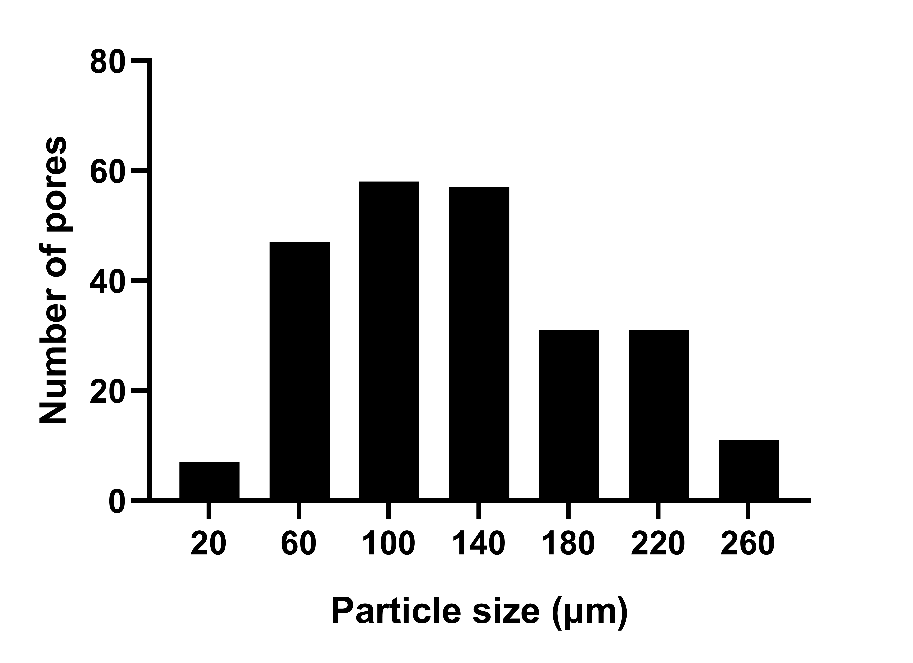


Figure S1 Pore size distribution of PCL/PDA/GE scaffold.


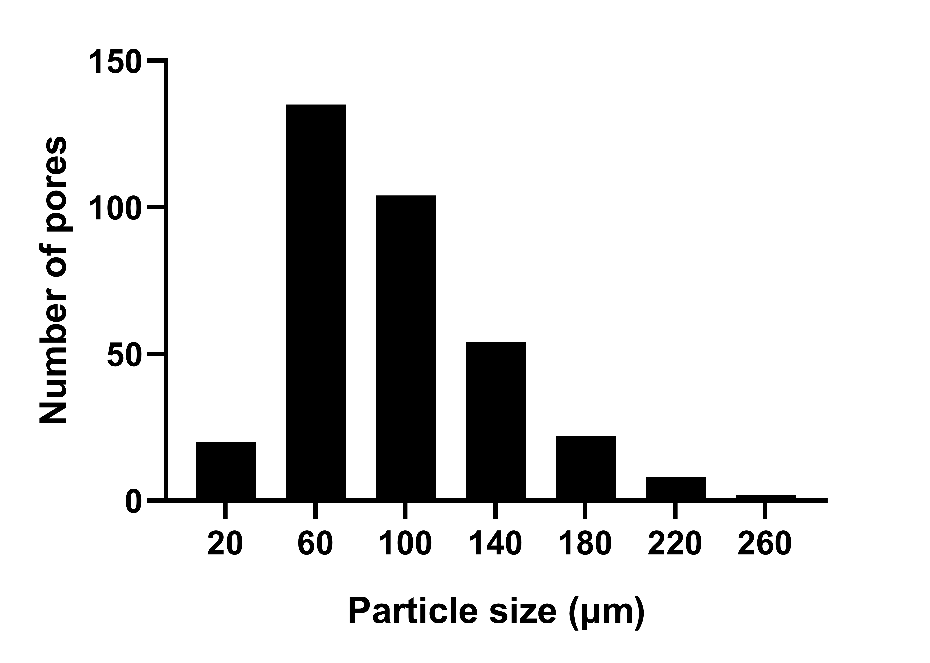


Figure S2 Pore size distribution of PCL/PDA/GE-Blank PLGA scaffold.


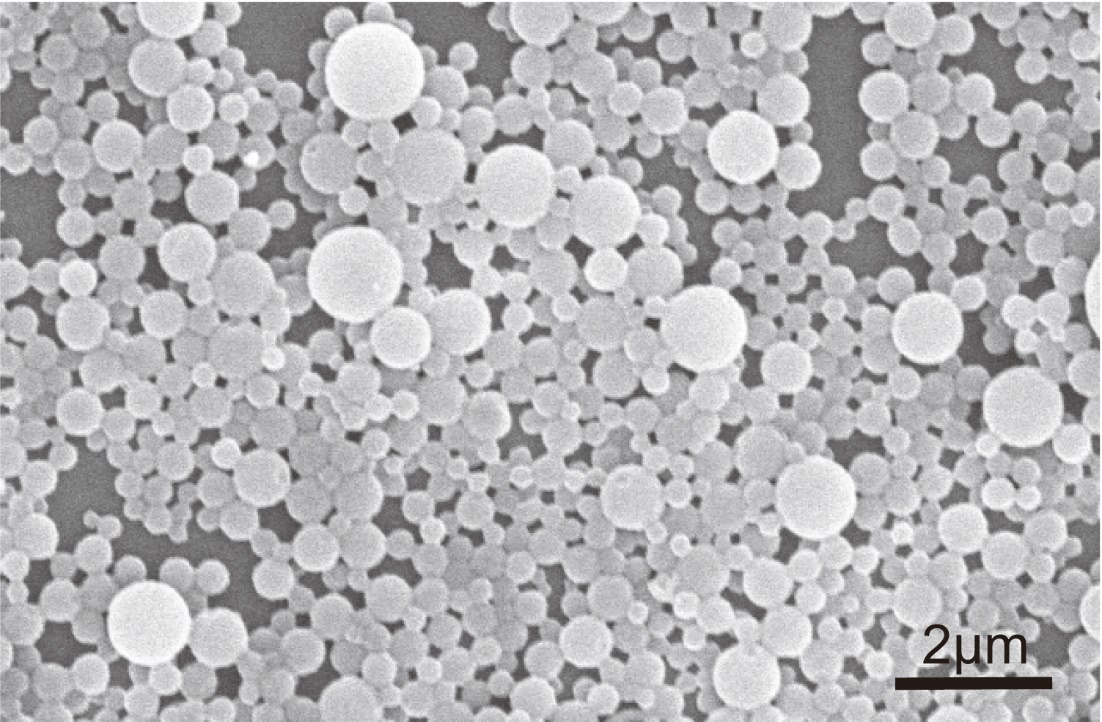


Figure S3 SEM image of PLGA nanoparticles (NPs).


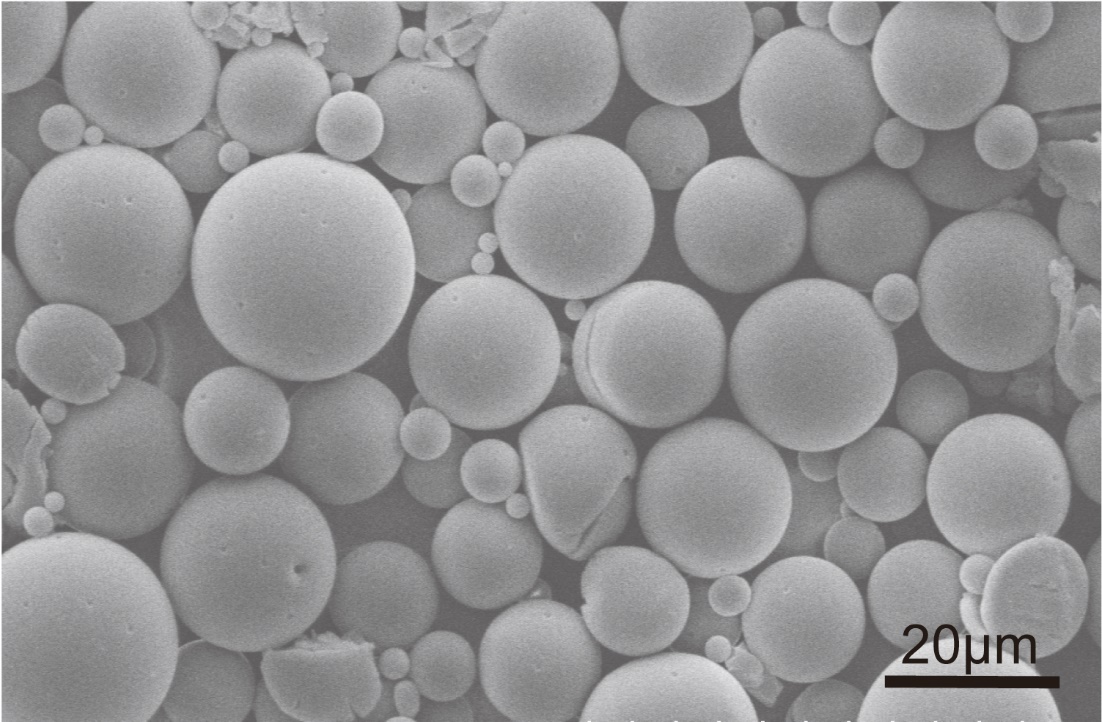


Figure S4 SEM image of PLGA microparticles (MPs).


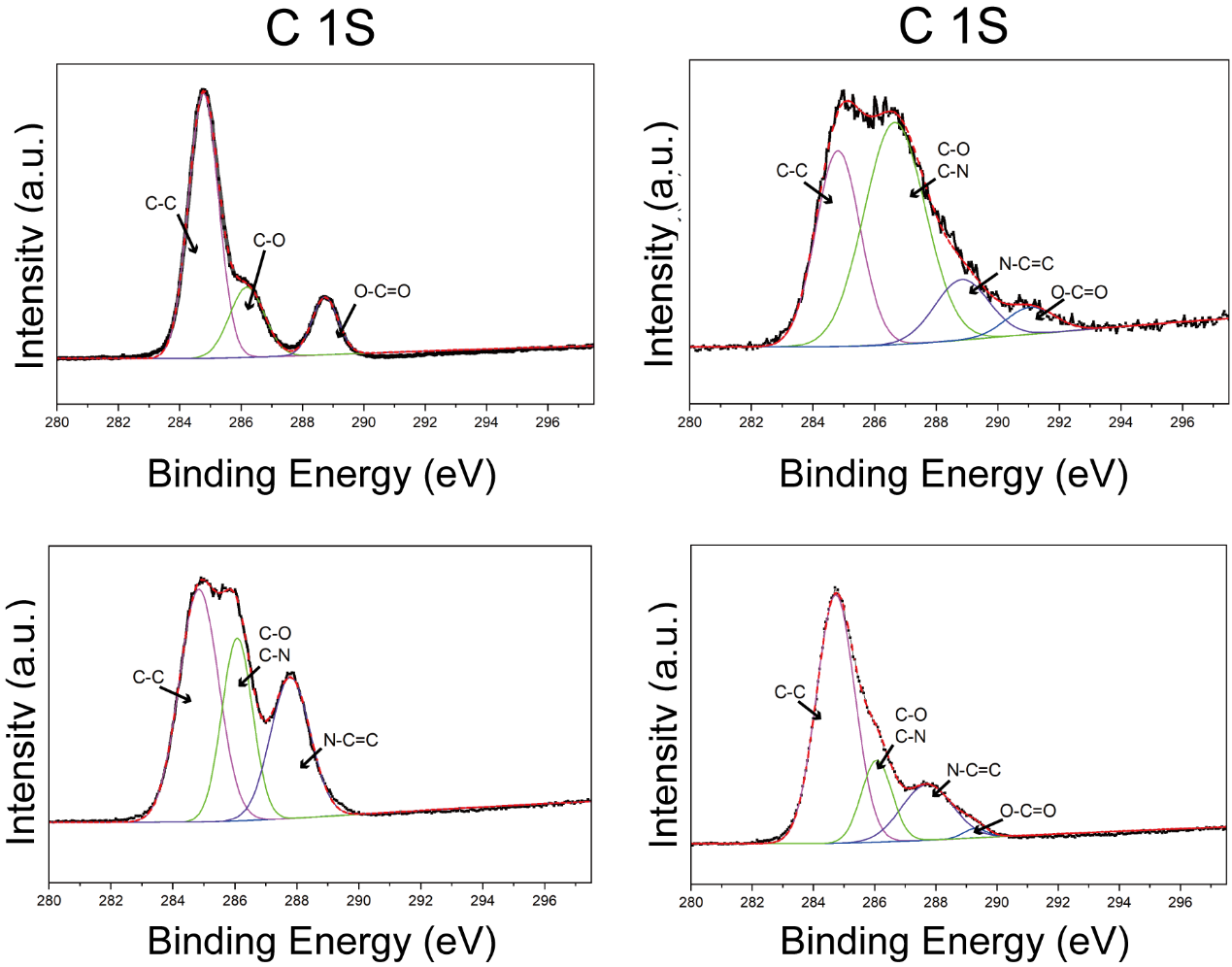


Figure S5 Three peaks at 289.0, 286.5 and 285.0 eV in the C 1s spectrum can be observed, which attributed to O-C=O, C-O and C-C groups of PCL, respectively[9]. With the introduction of PDA, two new peaks of C 1s appeared, one was N-C=O (288.57 eV), and the other was C-N (286.8 eV), which was overlapped with C-C. By further modification with GE and PLGA, there were no obvious new peaks in the C 1 s spectrum, while the intensity of each peak of the C1s mentioned above has changed.


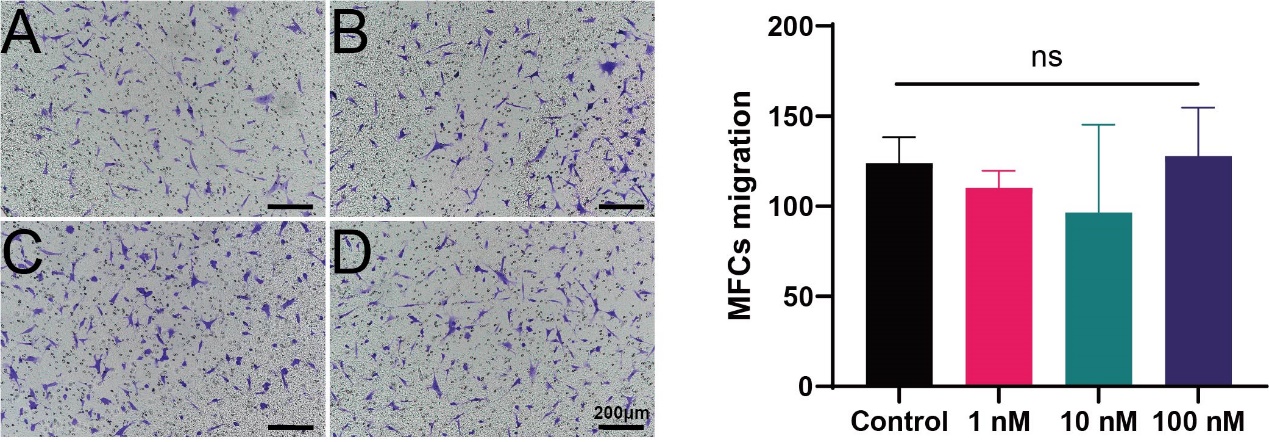


Figure S6 Statistical analysis and crystal staining (A-D) of MFCs migration toward the (A) control group, (B) 1 nM **Apt19S**, (C) 10 nM **Apt19S**, and (D) 100 nM **Apt19S** in a Transwell system (n=5). Data are means ± SD. ns, means no significant difference.


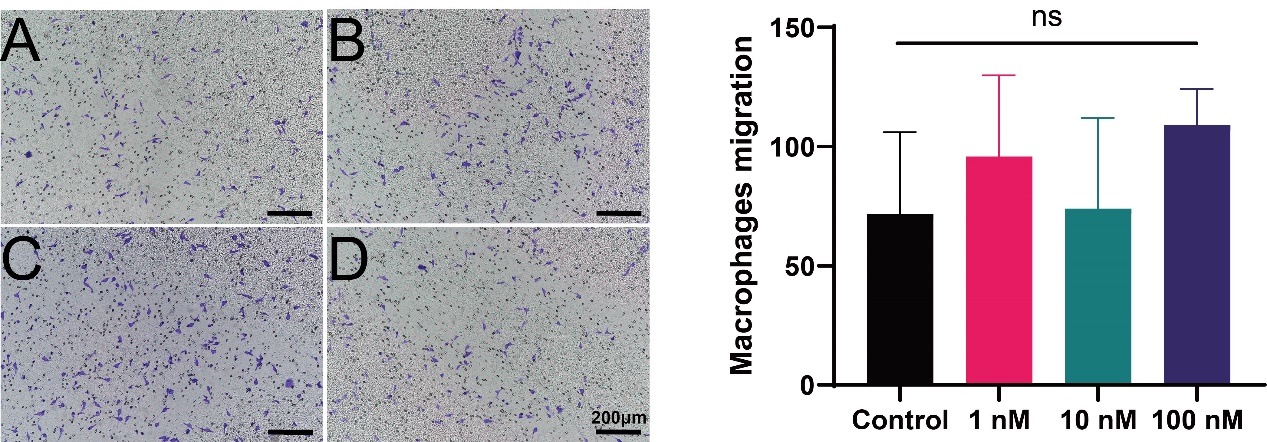


Figure S7 Statistical analysis and crystal staining (A-D) of macrophages migration toward the (A) control group, (B) 1 nM **Apt19S**, (C) 10 nM **Apt19S**, and (D) 100 nM **Apt19S** in a Transwell system (n=5). Data are means ± SD. ns, means no significant difference.


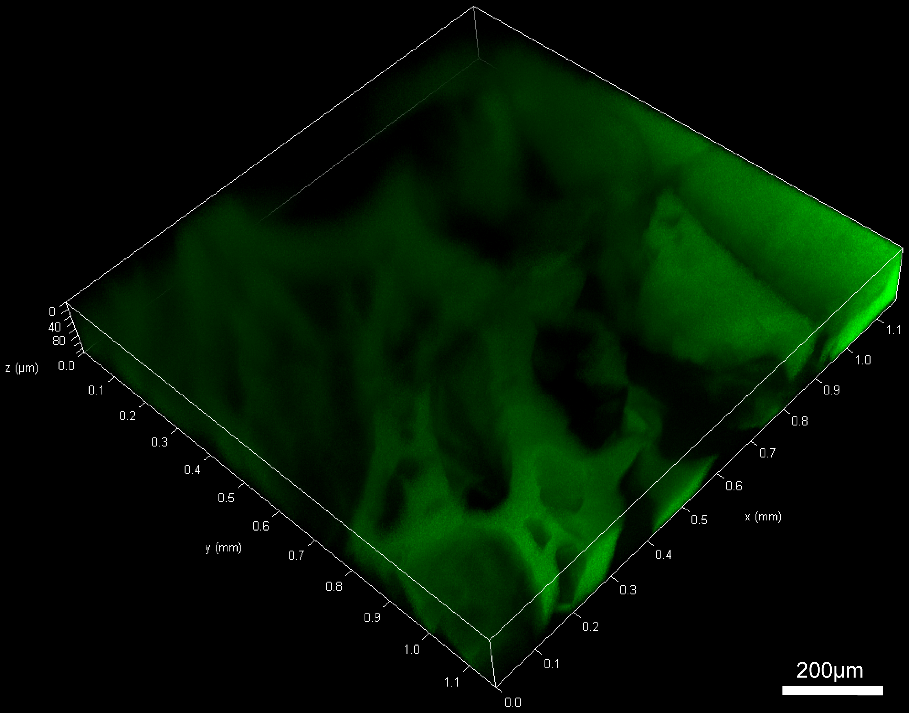


Figure S8 Confocal images of FAM-labeled **Apt19S** distribution in MECM sponge.

**Reference**

[1] Z. Yuan, S. Liu, C. Hao, W. Guo, S. Gao, M. Wang, M. Chen, Z. Sun, Y. Xu, Y. Wang, AMECM/DCB scaffold prompts successful total meniscus reconstruction in a rabbit total meniscectomy model, Biomaterials 111 (2016) 13-26.

[2] Z. Jian, T. Zhuang, T. Qinyu, P. Liqing, L. Kun, L. Xujiang, W. Diaodiao, Y. Zhen, J. Shuangpeng, S. Xiang, 3D bioprinting of a biomimetic meniscal scaffold for application in tissue engineering, Bioactive materials 6(6) (2021) 1711-1726.

[3] H. Li, Z. Liao, Z. Yang, C. Gao, L. Fu, P. Li, T. Zhao, F. Cao, W. Chen, Z. Yuan, 3D printed poly (ε-caprolactone)/meniscus extracellular matrix composite scaffold functionalized with kartogenin-releasing PLGA microspheres for meniscus tissue engineering, Frontiers in bioengineering and biotechnology 9 (2021) 282.

[4] P. Wei, Y. Xu, H. Zhang, L. Wang, Continued sustained insulin-releasing PLGA nanoparticles modified 3D-Printed PCL composite scaffolds for osteochondral repair, Chemical Engineering Journal 422 (2021) 130051.

[5] S. Tarafder, J. Gulko, K.H. Sim, J. Yang, J.L. Cook, C.H. Lee, Engineered healing of avascular meniscus tears by stem cell recruitment, Scientific reports 8(1) (2018) 1-9.

[6] Z. Li, N. Wu, J. Cheng, M. Sun, P. Yang, F. Zhao, J. Zhang, X. Duan, X. Fu, J. Zhang, Biomechanically, structurally and functionally meticulously tailored polycaprolactone/silk fibroin scaffold for meniscus regeneration, Theranostics 10(11) (2020) 5090.

[7] M. Chen, Z. Feng, W. Guo, D. Yang, S. Gao, Y. Li, S. Shen, Z. Yuan, B. Huang, Y. Zhang, PCL-MECM-Based hydrogel hybrid scaffolds and meniscal fibrochondrocytes promote whole meniscus regeneration in a rabbit meniscectomy model, ACS applied materials & interfaces 11(44) (2019) 41626-41639.

[8] Z. Yang, H. Li, Y. Tian, L. Fu, C. Gao, T. Zhao, F. Cao, Z. Liao, Z. Yuan, S. Liu, Biofunctionalized Structure and Ingredient Mimicking Scaffolds Achieving Recruitment and Chondrogenesis for Staged Cartilage Regeneration, Frontiers in cell and developmental biology 9 (2021) 680.

[9] J.S. Stevens, A.C. de Luca, S. Downes, G. Terenghi, S.L. Schroeder, Immobilisation of cell‐binding peptides on poly‐ε‐caprolactone (PCL) films: A comparative XPS study of two chemical surface functionalisation methods, Surface and Interface Analysis 46(10-11) (2014) 673-678.
